# Splicing regulation of GFPT1 muscle-specific isoform and its roles in glucose metabolisms and neuromuscular junction

**DOI:** 10.1016/j.isci.2023.107746

**Published:** 2023-08-26

**Authors:** Paniz Farshadyeganeh, Mohammad Nazim, Ruchen Zhang, Bisei Ohkawara, Kazuki Nakajima, Mohammad Alinoor Rahman, Farhana Nasrin, Mikako Ito, Jun-ichi Takeda, Kenji Ohe, Yuki Miyasaka, Tamio Ohno, Akio Masuda, Kinji Ohno

**Affiliations:** 1 Division of Neurogenetics, Center for Neurological Diseases and Cancer, Nagoya University Graduate School of Medicine, Nagoya 466-8550, Japan; 2 Department of Microbiology, Immunology, and Molecular Genetics, University of California, Los Angeles, Los Angeles, CA 90095, USA; 3 Institute for Glyco-core Research (iGCORE), Gifu University, Gifu 501-1193, Japan; 4 Department of Biochemistry and Molecular Biology, Winthrop P. Rockefeller Cancer Institute, University of Arkansas for Medical Sciences (UAMS), Little Rock, AR 72205, USA; 5 Faculty of Pharmaceutical Sciences, Fukuoka University, Fukuoka 814-0180, Japan; 6 Division of Experimental Animals, Nagoya University Graduate School of Medicine, Nagoya 466-8550, Japan

**Keywords:** Biological sciences, Biochemistry, Physiology

## Abstract

Glutamine:fructose-6-phosphate transaminase 1 (GFPT1) is the rate-limiting enzyme of the hexosamine biosynthetic pathway (HBP). A 54-bp exon 9 of *GFPT1* is specifically included in skeletal and cardiac muscles to generate a long isoform of GFPT1 (GFPT1-L). We showed that SRSF1 and Rbfox1/2 cooperatively enhance, and hnRNP H/F suppresses, the inclusion of human *GFPT1* exon 9 by modulating recruitment of U1 snRNP. Knockout (KO) of GFPT1-L in skeletal muscle markedly increased the amounts of GFPT1 and UDP-HexNAc, which subsequently suppressed the glycolytic pathway. Aged KO mice showed impaired insulin-mediated glucose uptake, as well as muscle weakness and fatigue likely due to abnormal formation and maintenance of the neuromuscular junction. Taken together, GFPT1-L is likely to be acquired in evolution in mammalian striated muscles to attenuate the HBP for efficient glycolytic energy production, insulin-mediated glucose uptake, and the formation and maintenance of the neuromuscular junction.

## Highlights

• Inclusion of *GFPT1* exon 9 in splicing makes GFPT1-L in mammalian striated muscles
• SRSF1 and Rbfox1/2 enhance, and hnRNP H/F suppresses, splicing of *GFPT1* exon 9
• Lack of GFPT1-L markedly increases GFPT1 and UDP-HexNAc in skeletal muscle
• Lack of GFPT1-L impairs glucose uptake and suppresses formation/maintenance of NMJ

## Graphical abstract

**Figure:**
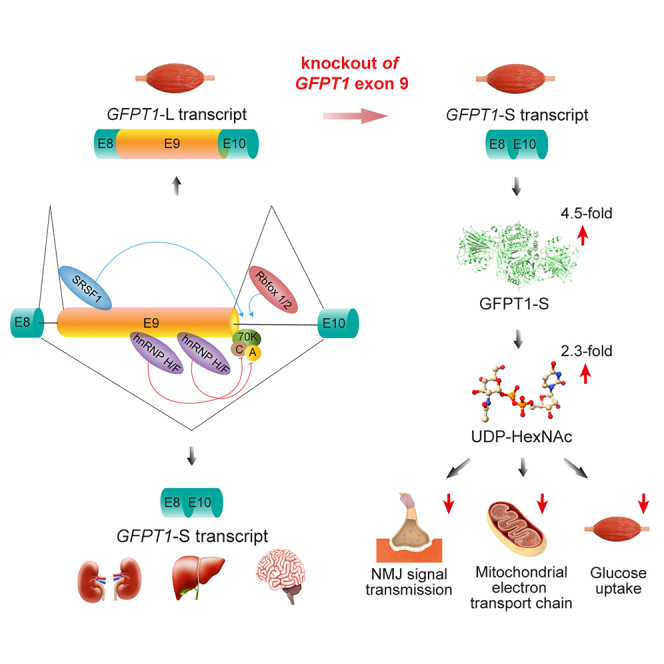

## Introduction

**Figure 1:**
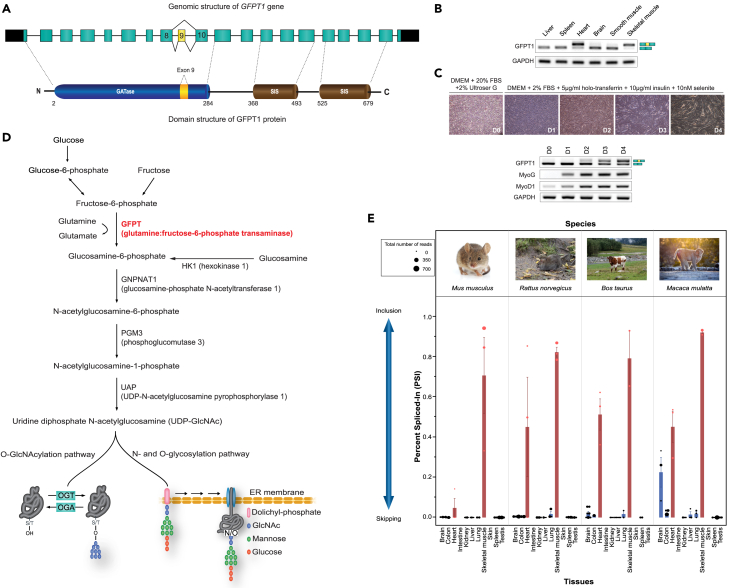
*GFPT1* exon 9 is specifically spliced-in in striated muscles only in mammals (A) Schematic of the genomic structure of human *GFPT1* gene and the domain structure of GFPT1 protein. Exons are shown in boxes and introns are shown in solid lines. The 5′ and 3′ untranslated regions (UTRs), constitutive exons, and alternatively spliced exon 9 are indicated in black, blue, and yellow, respectively. GATase, glutamine amidotransferase; SIS, sugar isomerase. (B) RT-PCR of endogenous *GFPT1* in human tissues. Splice variants are schematically shown on the right side. (C and D) Representative phase-contrast images of differentiating immortalized human KD3/Hu5 myoblasts. RT-PCR showing alternative splicing of endogenous *GFPT1* exon 9 at different days (D) of differentiating KD3/Hu5 myoblasts. Expressions of myogenin (MyoG), myogenic differentiation 1 (MyoD1) and GAPDH are shown as internal controls. (D) Schematic of the hexosamine biosynthetic pathway (HBP). A key regulator of the HBP, GFPT, catalyzes the first and rate-limiting step. OGT, O-GlcNAc transferase; OGA, O-GlcNAcase, DPAGT1, dolichyl-phosphate N-acetylglucosaminephosphotransferase 1; and ALGs, asparagine-linked glycosylation homologs. (E) Calculation of percent spliced-in (PSI) of *GFPT1*genes in 11 tissues in 4 mammals in RNA-seq in the public database. Note that *GFPT1* exon 9 and its flaking introns are conserved only in mammals (Figure S4). Skeletal muscle and heart are shown in red. Each PSI in public database is indicated by a circle, and the circle size represents the number of reads in RNA-seq. Mean and SD are indicated.

Hexosamine biosynthetic pathway (HBP) branches from the glycolysis pathway, and produces uridine diphosphate N-acetylglucosamine (UDP-GlcNAc) (Figure 1D). The rate of glucose consumption by HBP was quantified only in primary adipocytes derived from rat epididymal fat, which was as low as 2–3%.^1^ UDP-GlcNAc is the final product of HBP and is exclusively produced by HBP. UDP-GlcNAc is utilized for N- and O-linked glycosylation of glycoproteins, as well as for the synthesis of glycosaminoglycans and glycolipids. UDP-GlcNAc also modulates signaling pathways by O-GlcNAcylation of signaling molecules, and is a metabolic regulator for both stress response and nutrient sensing.^2^^,^^3^ UDP-GlcNAc and UDP-GalNAc are interconvertible by UDP-GlcNAc 4-epimerase, and they are collectively called UDP-HexNAc. Glutamine:fructose-6-phosphate transaminase (GFPT/GFAT) is the first-step and rate-limiting enzyme of HBP, which catalyzes the conversion of fructose-6-phosphate (F-6-P) to glucosamine-6-phosphate (GlcN-6-P), as well as glutamine to glutamate in parallel, via an amidotransaminase reaction (Figure 1D).^4^ Paralogous GFPT1 and GFPT2 are comprised 681 and 682 amino acids in humans, respectively.^5^ GFPT1 and GFPT2 are ∼76% identical at the amino acid level in both humans and mice.^5^ According to the GTEx project,^6^ both *GFPT1* and *GFPT2* are widely expressed in all human tissues, but their levels are variable from tissue to tissue.^5^^,^^7^ For example, in human skeletal muscle, the expression level of *GFPT1* is 5.20 times higher than that of *GFPT2* in the GTEx database. Alternative splicing of *GFPT1* exon 9 generates a short ubiquitous isoform (GFPT1-S) and a long muscle-specific isoform (GFPT1-L) (Figure S1A).^8^^,^^9^ In humans and mice, GFPT1-L is exclusively expressed in skeletal muscle and is expressed along with GFPT1-S in the heart, but not in the other tissues where GFPT1-S is exclusively expressed (Figure S1B).^8^^,^^9^ *GFPT1* exon 9 (54 bp) encodes 18 amino acids (54 bp) in humans and 16 amino acids (48 bp) in mice. GFPT1-L has a lower enzymatic activity compared to GFPT1-S.^8^^,^^9^ GFPT1-L receives a higher feedback inhibition by UDP-GlcNAc than GFPT1-S.^8^^,^^9^ However, the splicing regulation and the physiological significance of GFPT1-L in skeletal muscle remain unelucidated.

Pathogenic variants of *GFPT1* have been reported in congenital myasthenic syndrome (CMS).^10^^,^^11^ *GFPT1*-CMS patients exhibit progressive limb-girdle muscle weakness, and have poorly developed junctional folds at the neuromuscular junction (NMJ).^12^^,^^13^^,^^14^^,^^15^^,^^16^ Muscle-specific *Gfpt1*-deficient mouse exhibits muscle weakness, abnormal fatigue, presynaptic and post synaptic remodeling of the NMJ with fewer junctional folds, and small and fragmented acetylcholine receptor (AChR) clusters.^17^ Pathogenic variants in genes encoding enzymes in N-linked glycosylation (*ALG2*, *ALG14*, and *DPAGT1*) also cause limb-girdle CMS, suggesting the involvement of HBP and UDP-GlcNAc in the formation and maintenance of the NMJ.^18^^,^^19^^,^^20^^,^^21^^,^^22^ Glucosamine is converted to glucosamine-6-phosphate by hexokinase-1, and increases the production of UDP-GlcNAc without utilizing GFPT1 (Figure 1D). Increased production of UDP-GlcNAc by glucosamine also induces insulin resistance in skeletal muscle.^23^^,^^24^ Similarly, the enzymatic activity of GFPT1 is elevated in skeletal muscle in patients with non-insulin dependent diabetes mellitus (NIDDM).^25^ Conversely, transgenic mice overexpressing *Gfpt1* in skeletal muscle and fat develop insulin resistance, which is a hallmark of NIDDM.^26^^,^^27^

SRSF1 is a ubiquitously expressed RBP of the serine- and arginine-rich (SR) protein family. SRSF1 primarily promotes exon inclusion by binding to an exonic splicing enhancer in both constitutively and alternatively spliced exons.^28^ The Rbfox proteins are brain- and muscle/heart-specific RBPs, and are comprised paralogous Rbfox1, Rbfox2, and Rbfox3. While Rbfox1 and Rbfox2 are preferentially expressed in neurons, skeletal muscle, and heart, Rbfox3 is highly expressed in post-mitotic neurons.^29^^,^^30^^,^^31^ Rbfox proteins bind to the highly conserved (U)GCAUG element across all vertebrate species.^29^^,^^30^^,^^32^^,^^33^ The binding of Rbfox to an alternative exon or its upstream intron represses splicing, whereas the binding to its downstream intron activates splicing.^29^^,^^30^ HnRNP H and hnRNP F are closely related and ubiquitously expressed RBPs that belong to the heterogeneous nuclear ribonucleoprotein (hnRNP) family.^34^^,^^35^ HnRNP H and hnRNP F share highly conserved sequences (68% amino acid identity), and both bind to poly(G)-rich sequences (G-runs) located in a target exon and its flanking introns to regulate alternative splicing.^36^^,^^37^ We and others have shown that these proteins also function as regulators of alternative polyadenylation.^35^^,^^38^^,^^39^^,^^40^^,^^41^

We here dissected the molecular mechanism of muscle-specific alternative splicing of exon 9 in human *GFPT1*. We showed that SRSF1, Rbfox1/2, and hnRNP H/F cooperatively regulated the alternative splicing of human *GFPT1* exon 9. To understand the functional significance of GFPT1-L in skeletal muscle, we generated *Gfpt1* exon 9 knockout (KO) mouse and found that lack of *Gfpt1* exon 9 markedly increased GFPT1-S and UDP-GlcNAc, and compromised glycolytic energy production, insulin-mediated glucose uptake, and the formation and maintenance of the NMJ.

## Results

### Muscle-specific *GFPT1* exon 9 is alternatively spliced in skeletal muscle and heart in mammals

The inclusion of exon 9 in humans incorporates 18 amino acids close to the C-terminal end of the glutamine amidotransferase (GATase) domain of GFPT1 protein (Figure 1A) and alters its enzymatic activity.^8^^,^^9^ In agreement with previous reports,^8^^,^^9^ RT-PCR of total RNA extracted from various human tissues showed that *GFPT1* exon 9 was included only in skeletal muscle and heart (Figure 1B). Similar alternative inclusion of exon 9 was observed in the course of myotube differentiation of immortalized human KD3/Hu5 myoblasts (Figure 1C).

The phylogenetic tree of paralogous *GFPT1* and *GFPT2* by Ensembl showed that they were likely to have evolved after the appearance of vertebrates, because invertebrate *GFPT1/2*, vertebrate *GFPT1*, and vertebrate *GFPT2* make distinct clusters (Figure S2). The HomoloGene database at NCBI also showed that invertebrate *GFPT1*/*2* is likely to be ancestor(s) of vertebrate *GFPT2*, but not vertebrate *GFPT1* (Figure S3). Multiple alignment of genomic sequences of *GFPT1* exon 9 in 100 vertebrates using the UCSC genome browser showed that amino acid sequences encoded by exon 9 are highly conserved in mammals (Figure S4). We calculated tissue-specific percent spliced-in (PSI) of *GFPT1* exon 9 in four mammals, and found that exon 9 was exclusively included in skeletal muscle and heart (red bars in Figure 1E).

### RNA-binding proteins, SRSF1, Rbfox1/2, and hnRNP H/F, regulate alternative splicing of *GFPT1* exon 9

**Figure 2:**
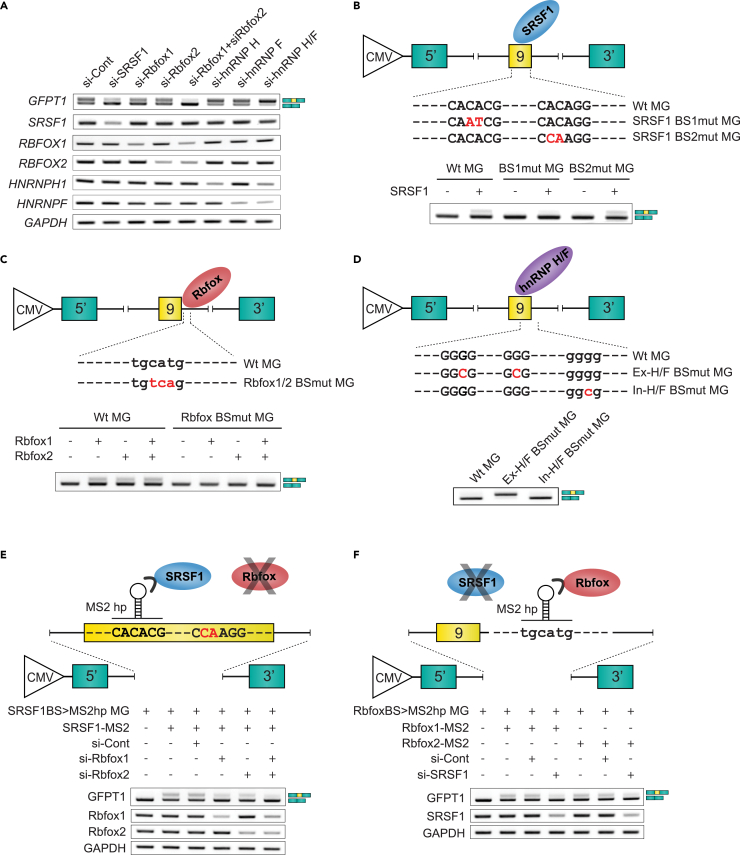
RNA-binding proteins, SRSF1, Rbfox1/2, and hnRNPs regulate alternative splicing of *GFPT1* exon 9 (A) RT-PCR of endogenous *GFPT1* in differentiated KD3/Hu5 myoblasts after introduction of siRNAs against control (si-Cont), *SRSF1* (si-SRSF1), *RBFOX1* (si-Rbfox1), *RBFOX2* (si-Rbfox2), *HNRNPH1* (si-hnRNP H), *HNRNPF* (si-hnRNP F), and both *HNRNPH1* and *HNRNPF* (si-hnRNP H/F). Splicing variants of *GFPT1* exon 9 are schematically shown on the right side. (B and C) RT-PCR of KD3/Hu5 myoblasts transfected with *GFPT1* minigenes (MG) carrying wild type (WT MG) and mutant binding sites for SRSF1 (SRSF1 BS1mut MG and SRSF1 BS2mut MG) (B) and Rbfox1/2 (Rbfox1/2 BSmut MG) (C) in the presence and absence of overexpression of SRSF1 (B) and Rbfox1/2 (C), respectively. (D) RT-PCR of KD3/Hu5 myoblasts transfected with *GFPT1* minigenes carrying wild type (WT MG) and mutant exonic (Ex-H/F BSmut MG) and intronic (In-H/F BSmut MG) binding sites for hnRNP H/F. (E) Schematic of the *GFPT1* minigene carrying the MS2 hairpin-loop (hp) substituting for the first SRSF1-binding motif in exon 9 (SRSF1BS > MS2hp MG), while the second putative SRSF1-binding motif is mutated (red letters). SRSF1 is fused to the MS2 coat protein (SRSF1-MS2) (inverted U-shape) to directly tether SRSF1 to the MS2 hairpin-loop with or without knocking down of Rbfox1 and Rbfox2. (F) Schematic of the *GFPT1* minigene carrying the MS2 hairpin-loop substituting for the Rbfox-binding motif in intron 9 (RbfoxBS>MS2hp MG). Rbfox1 and Rbfox2 are fused to the MS2 coat protein (Rbfox1-MS2 and Rbfox2-MS2) to directly tether Rbfox1 and Rbfox2 to the MS2 hairpin-loop, respectively, with or without knocking down of SRSF1. (A–F) In all the transfection studies, minigenes, siRNAs, and RNA-binding proteins were transfected into KD3/Hu5 myoblasts on differentiation day 1 and harvested on differentiation day 3.

Putative binding motifs of RNA-binding proteins (RBPs) on human *GFPT1* exon 9 and its flanking introns were searched for using human splicing factor databases, ESE finder 3.0^42^ and SpliceAid 2^43^ (Figure S5A). Knocking down of candidate RBPs revealed that downregulation of SRSF1 causes skipping of exon 9 in differentiating human KD3/Hu5 myotubes (Figure 2A). Similarly, downregulation of Rbfox1 partially and Rbfox2 marginally enhanced skipping of exon 9, while simultaneous downregulation of both Rbfox1 and Rbfox2 almost exclusively skipped exon 9, indicating that these RBPs are functionally redundant to each other (Figure 2A). In contrast, individual downregulation of hnRNP H or F partially enhanced inclusion of exon 9, whereas simultaneous downregulation of both hnRNP H and F resulted in almost exclusive inclusion of exon 9 (Figure 2A). Downregulation of the other candidate RBPs (SRSF2, SRSF3, SRSF5, YB1, hnRNP A1) had marginal effects on splicing (Figure S5B). Taken together, SRSF1, Rbfox1, and Rbfox2 functioned as splicing enhancers, whereas hnRNP H and F functioned as splicing silencers for *GFPT1* exon 9.

To dissect splicing *cis*-elements for *GFPT1* exon 9, we constructed human *GFPT1* minigene by inserting exon 9 and its flanking introns in the modified exon-trapping pSPL3 vector.^44^^,^^45^ As has been reported in other minigenes,^35^^,^^46^^,^^47^ splicing efficiency of the pSPL3 minigene was low and exon 9 was not included in transfected KD3/Hu5 myoblasts/myotubes (Figures 2B–2D). ESE finder predicted two high-affinity binding motifs for SRSF1 in exon 9, with the sequences of CACACGG (score 5.86) and CACAGGG (score 5.25). RT-PCR showed that overexpression of SRSF1 partially increased exon 9 inclusion in the wild-type *GFPT1* minigene (Figure 2B). In contrast, disruption of the first putative SRSF1-binding motif (CACACGG) compromised SRSF1-mediated exon 9 inclusion, whereas disruption of the second putative SRSF1 binding motif (CACACGG) had no such effect (Figure 2B), indicating that the first binding motif is the functional motif for SRSF1. Since disruption of the second putative SRSF1-binding motif had no effect on splicing, we did not make a construct that disrupted both putative SRSF1 binding motifs. It is well established that Rbfox proteins bind to the (U)GCAUG motif.^29^^,^^48^ An Rbfox motif of UGCAUG is present in intron 9. Overexpression of either Rbfox1 or Rbfox2 enhanced exon 9 inclusion in the wild-type *GFPT1* minigene (Figure 2C). In contrast, disruption of the Rbfox-binding motif abrogated exon 9 inclusion even with overexpression of Rbfox1 and/or Rbfox2, indicating that the intronic UGCAUG motif is the target binding site of Rbfox1/2 (Figure 2C). Paralogous proteins hnRNP H and F bind to G-runs to regulate alternative splicing.^35^^,^^36^^,^^37^ There are two G-runs in exon 9 and one G-run in intron 9. Disruption of the exonic G-runs resulted in exclusive inclusion of exon 9, whereas disruption of the intronic G-run had no such effect, indicating that the exonic G-runs are functional motifs for hnRNP H and F (Figure 2D).

To further identify the position-specific effects of the identified *trans-*acting splicing factors, we tethered each RBP to a specific RNA segment using the bacteriophage coat proteins MS2 or PP7. To this end, SRSF1, Rbfox1, and Rbfox2 were fused with MS2 coat protein (SRSF1-MS2, Rbfox1-MS2, and Rbfox2-MS2), and hnRNP H and F were fused with PP7 coat protein (hnRNP H-PP7 and hnRNP F-PP7) (Figure S6). We first introduced the MS2 coat protein-binding hairpin-loop sequence (MS2-HP) in place of the first SRSF1 binding motif (CACACGG) in exon 9, while the second putative SRSF1 binding motif was mutated (CCAAGGG). As expected, tethering of SRSF1 induced exon 9 inclusion (Figure S7A). We next replaced the Rbfox binding motif (UGCAUG) with the MS2-HP, and found that tethering of either Rbfox1 or Rbfox2 enhanced exon 9 inclusion (Figure S7B). We then replaced the two exonic G-runs in exon 9 (GGGG … GGG) with the PP7 coat protein-binding hairpin-loop sequence (PP7-HP). As predicted, tethering of either hnRNP H or hnRNP F suppressed exon 9 inclusion (Figure S7C). Thus, our data demonstrate that the splicing regulatory RBPs function through binding to their specific *cis*-regulatory motifs to regulate *GFPT1* exon 9 splicing.

We next questioned whether SRSF1 and Rbfox1/2 cooperatively enhance exon 9 inclusion. As shown previously, tethering of SRSF1 enhanced exon 9 inclusion; however, individual knockdown of Rbfox1 or Rbfox2 partially suppressed the effect of SRSF1 (Figure 2E). In addition, simultaneous knockdown of both Rbfox1 and Rbfox2 markedly suppressed the effect of SRSF1. Similarly, tethering of either Rbfox1 or Rbfox2 enhanced exon 9 inclusion, but simultaneous knockdown of SRSF1 suppressed these effects (Figure 2F). Thus, SRSF1 and Rbfox1/2 depend on each other for efficient inclusion of *GFPT1* exon 9.

### SRSF1 and Rbfox1/2 enhance U1 snRNP recruitment at the 5′ splice site, whereas hnRNP H/F suppress the recruitment

**Figure 3:**
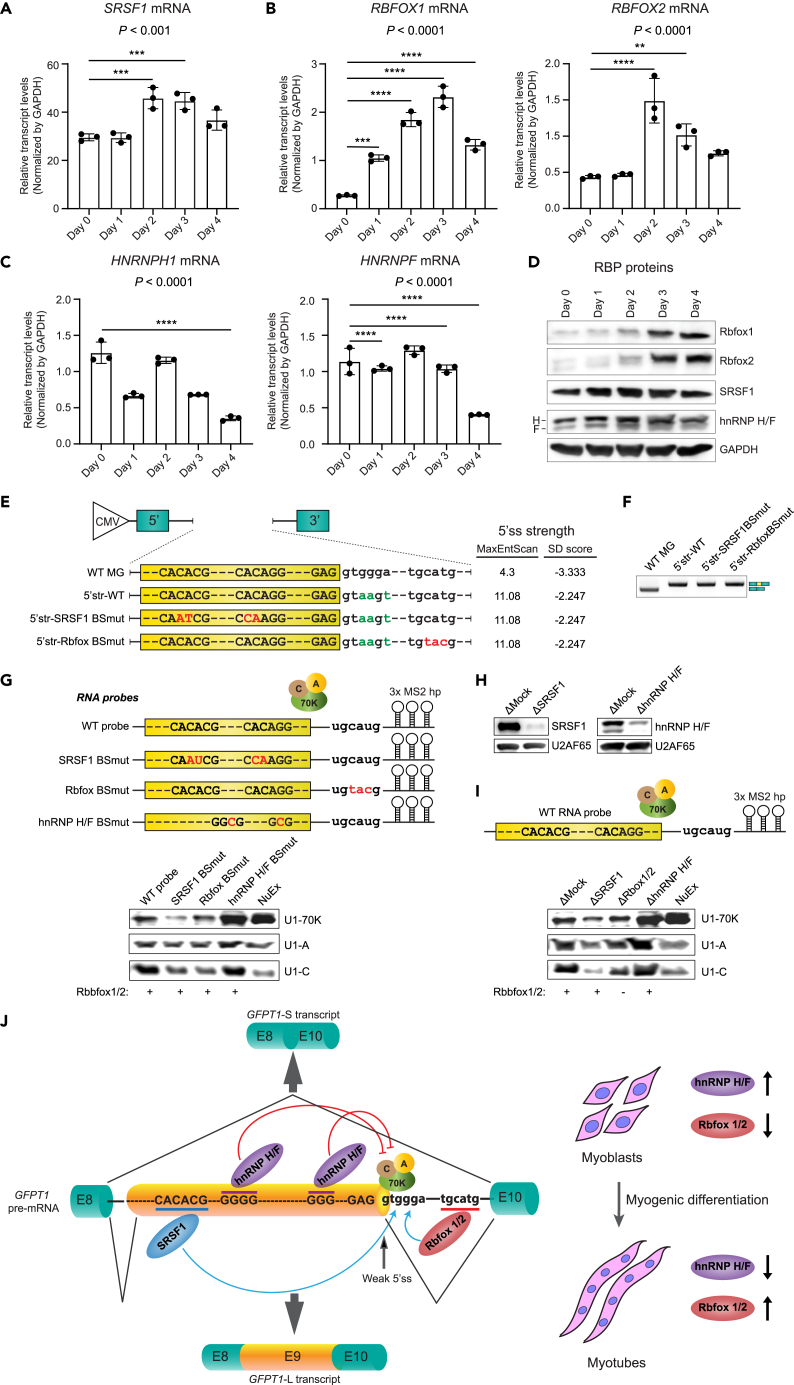
SRSF1 and Rbfox1/2 enhance, and hnRNP H/F suppress, the recruitment of U1 snRNP components at the 5′ splice site of *GFPT1* exon 9 (A–C) qRT-PCR to quantify transcripts for (A) *SRSF1*, (B) *RBFOX1* and *RBFOX2*, and (C) *HNRPH1* and *HNRNPF* in the course of myogenic differentiation of KD3/Hu5 myoblasts. Mean and SD are indicated (n = 3). p values by one-way ANOVA are indicated below the gene names. ∗p < 0.05, ∗∗p < 0.01, ∗∗∗p < 0.001, and ∗∗∗∗p < 0.0001 by Sidak posthoc test. (D) Immunoblots of endogenous SRSF1, Rbfox1, Rbfox2, hnRNP H, and hnRNP F in the course of myogenic differentiation of KD3/Hu5 myoblasts. (E) Schematic of *GFPT1* minigenes carrying wild type (WT) or artificially optimized 5′ splice site (green letters) at the exon 9/intro 9 boundary. Additional mutations (shown in red letters) were introduced to disrupt the binding sites for either SRSF1 or Rbfox1/2. Respective MaxEntScan::score5ss^49^ and SD scores^50^ at the 5′ splice sites are indicated on the right. (F) RT-PCR of minigene constructs shown in (E) transfected in KD3/Hu5 myoblasts. (G) Schematic of 3xMS2 hairpin-attached wild type and mutant RNA probes used for the pull-down of early spliceosome complex. In the mutant probes, the binding sites for SRSF1, Rbfox, and hnRNP H/F were disrupted each. Recombinant Rbfox1 and Rbfox2 were supplemented to the splicing-competent HeLa nuclear extract. Immunoblotting of the U1 snRNP components (U1-70K, U1A, and U1C) assembled on the indicated RNA probes are shown in the bottom panel. (H) Immunoblots of HeLa nuclear extracts depleted for Mock (ΔMock), SRSF1 (ΔSRSF1), and hnRNP H/F (ΔhnRNP H/F). U2AF65 was used as an internal control. (I) Schematic of 3xMS2 hairpin-attached wild-type RNA probe used for the pull-down of early spliceosome complex. As neither Rbfox1 nor Rbfox2 was detected in HeLa nuclear extracts, recombinant Rbfox1 and Rbfox2 were supplemented to the splicing-competent HeLa nuclear extract except for lane 3, which was labeled as ΔRbfox1/2. Immunoblotting of the U1 snRNP components assembled on the wild-type RNA probe with nuclear extracts depleted for the indicated proteins are shown in the bottom panel. (J) Model of coordinated tissue-specific alternative splicing regulation of *GFPT1* exon 9 mediated by SRSF1, Rbfox1/2, and hnRNP H/F. In the undifferentiated myoblasts, expression levels of hnRNP H/F are high, with very low expression levels of Rbfox1 and Rbfox2. In the lack of Rbfox1 and Rbfox2, SRSF1 alone fails to recruit U1 snRNP at the weak 5′ splice site (ss) at the exon 9/intron 9 border. Additionally, high expression levels of hnRNPs H and F suppress the recruitment of U1 snRNP at the weak 5′ splice site and produce the GFPT1 short (*GFPT1*-S) transcript. On the contrary, Rbfox1 and Rbfox2 expression levels are high in differentiated myotubes, with lower expression levels of hnRNPs H and F. SRSF1 and Rbfox1/2 cooperatively enhance the recruitment of U1 snRNP at the weak 5′ splice site, and thereby ensure the inclusion of exon 9 to produce *GFPT1-L* transcript in differentiated myotubes.

We next examined the expression levels of the identified *trans-*acting RBPs in myotube differentiation of human KD3/Hu5 myoblasts. qRT-PCR showed that SRSF1 was highly expressed in undifferentiated myoblasts and was marginally increased upon myotube differentiation (Figure 3A). Similarly, the expression levels of Rbfox1 and Rbfox2 were markedly increased during myotube differentiation (Figure 3B). In contrast, the expression levels of hnRNP H and hnRNP F tended to decrease upon myotube differentiation (Figure 3C). Similar results were observed at the protein level by western blotting in the course of myotube differentiation of KD3/Hu5 myoblasts (Figure 3D).

As the alternative inclusion of *GFPT1* exon 9 is specific to mammals, we next asked whether the binding sites of SRSF1, Rbfox1/2, and hnRNP H/F were conserved across species. Alignment of the genome sequences revealed that Rbfox1/2-binding site (UGCAUG) in intron 9, as well as the first SRSF1-binding site (CACACGG) and two hnRNP H/F-binding sites (GGG) in exon 9, were conserved in mammals but not in other species (Figure S4). Thus, the splicing regulation of *GFPT1* exon 9 by SRSF1, Rbfox1/2, and hnRNP H/F is likely to be conserved across mammalian species.

We noticed that the 5′ splice site at the boundary of exon 9 and intron 9 had a weak splicing *cis*-element with the GAG|gtggga sequence with a MaxEntScan score^49^ of 4.3 and an SD score^50^ of −3.333. We then made a minigene construct carrying the optimal 5′ splice site sequence (GAG|gtaagt) with a MaxEntScan score of 11.08, and an SD score of −2.247 (Figure 3E). RT-PCR showed that exon 9 is exclusively included when the 5′ splice site was optimally strengthened (Figure 3F). In addition, exon 9 was not skipped even when the SRSF1- or Rbfox-binding site was mutated in the optimally strengthened minigene construct, indicating that the optimization of the 5′ splice site made SRSF1 and Rbfox1/2 dispensable (Figure 3F). Thus, the weak 5′ splice site makes *GFPT1* exon 9 being alternatively spliced and being regulated by splicing *trans*-factors, SRSF1, Rbfox1/2, and hnRNP H/F.

We next analyzed the assembly of U1 snRNPs at the 5′ splice site in the presence or absence of SRSF1, Rbfox1/2, and hnRNP H/F. We first made four different RNA probes, each harboring exon 9 and its flanking intronic regions attached to a 3xMS2 hairpin loop sequence for pull-down assays. The first probe had the wild-type sequence, whereas the three other probes carried mutations disrupting SRSF1-, Rbfox1/2-, and hnRNP H/F-binding sites, respectively (Figure 3G). We incubated the RNA probes on the MS2 coat protein-coated beads with splicing-competent HeLa nuclear extract to assemble and pull down the early spliceosome complex at the 5′ splice site. The HeLa nuclear extract was supplemented with recombinant human Rbfox1 and Rbfox2 (10 ng/μL each), since we could not observe any detectable expression for these proteins in the HeLa nuclear extract. We observed that the association of U1 snRNP proteins (U1-70K, U1A, and U1C) to the RNA probe was markedly reduced when SRSF1- and Rbfox1/2-binding sites were mutated, and markedly enhanced when hnRNP H/F-binding sites were mutated (Figure 3G).

To further dissect the mechanism of U1 snRNP recruitment at the 5′ splice site, we next depleted each regulatory RBP from the HeLa nuclear extract (Figure 3H), and performed the U1 snRNP pull-down assay using these extracts. Since we could not observe any detectable expression of Rbfox1 and Rbfox2 in HeLa nuclear extract, we added recombinant Rbfox1 and Rbfox2 in the mock-depleted (ΔMock), SRSF1-depleted (ΔSRSF1), and hnRNP H/F-depleted (ΔhnRNP H/F) nuclear extracts. Mock-depleted HeLa extracts without adding recombinant Rbfox1 and Rbfox2 were considered as Rbfox-depleted (ΔRbfox1/2) nuclear extract. We incubated the wild-type RNA probe with the nuclear extracts and examined the assembly of early spliceosome complex at the 5′ splice site. As expected, the recruitment of U1 snRNP molecules was markedly reduced in ΔSRSF1 and ΔRbfox1/2 nuclear extracts, whereas the recruitment was further enhanced in ΔhnRNP H/F extract, compared to ΔMock nuclear extract (Figure 3I). Taken together, SRSF1 and Rbfox1/2 enhance, and hnRNP H/F suppresses, U1 snRNP recruitment at the 5′ splice site.

### Lack of GFPT1-L in skeletal muscle markedly increases GFPT1-S and UDP-GlcNAc, and attenuates glycolysis and TCA cycle, as well as glucose uptake in response to insulin, in skeletal muscle

**Figure 4:**
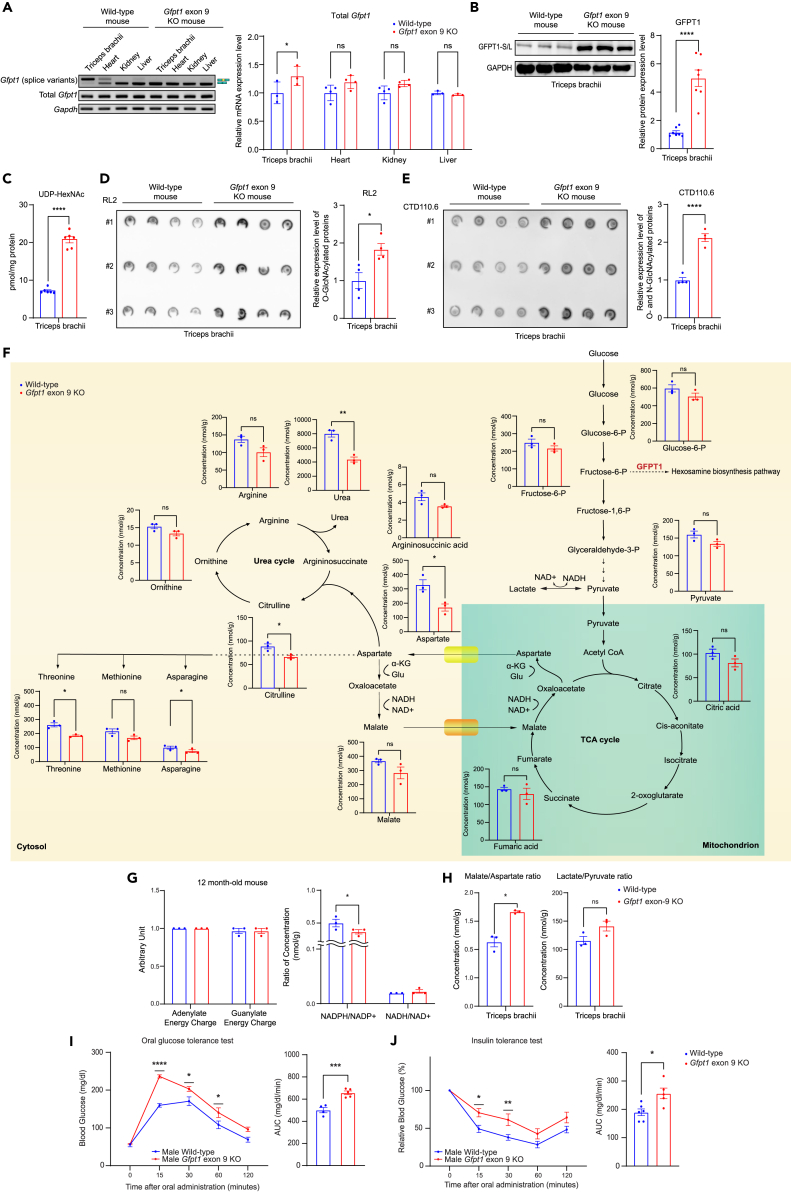
*Gfpt1* exon 9 is required for down-regulation of O-GlcNAcylation and N-glycosylation in skeletal muscle to regulate glucose uptake in response to insulin (A) Representative and quantitative RT-PCR of endogenous *Gfpt1* in indicated tissues of wild type and *Gfpt1* exon 9 KO mice at age 12 months (n = 4 mice each). Splice variants are schematically shown on the right side. (B) Representative immunoblots and quantification of endogenous Gfpt1 (both short and long isoforms, S/L) in triceps brachii of wild type and *Gfpt1* exon 9 KO mice at age 12 months (n = 7 mice each). (C) LC/MS/MS analysis of UDP-HexNAc (UDP-GlcNAc plus UDP-GalNAc) in triceps brachii of wild type and *Gfpt1* exon 9 KO mice (n = 6 mice each). (D) Triplicated blots and quantification of O-GlcNAcylated proteins probed with RL2 antibody in triceps brachii of wild-type and *Gfpt1* exon 9 KO mice (n = 4 mice each). (E) Triplicated blots and quantification of O-GlcNAcylated and N-glycosylated proteins probed with CTD110.6 antibody in triceps brachii of wild type and *Gfpt1* exon 9 KO mice (n = 4 mice each). (F) Metabolomic analysis of triceps brachii of wild type and *Gfpt1* exon 9 KO mice at age 13 months (n = 3 mice each). Pathway diagram of glycolysis, TCA cycle, and urea cycle is indicated with the concentration of each metabolite. (G) Intracellular energy status (adenylate and guanylate energy charges) and the redox status (NADPH/NADP^+^ and NADH/NAD^+^ ratios) were calculated using the metabolomic data (Table S2) (n = 3 mice each). (H) The malate/aspartate and lactate/pyruvate ratios, which are indirect indicators for NADH/NAD^+^, were calculated using the metabolomic data (Table S2) (n = 3 mice each). (I and J) Glucose tolerance test of wild-type mice (n = 4) and *Gfpt1* exon 9 KO mice (n = 5) at age 12 months. The area under the curves (AUCs) are plotted on the right. (J) Insulin tolerance tests of wild-type mice (n = 6) and *Gfpt1* exon 9 KO mice (n = 5) at age 12 months. The AUCs are plotted on the right. (A–J) Mean and SEM are indicated. Unpaired Student’s *t* test was applied to (B, C, D, E, H, I) (AUC), and J (AUC). Welch’s *t*-test was applied to F. two-way ANOVA with posthoc Tukey test was applied to (G and I) (temporal profile), and J (temporal profile). ∗p < 0.05, ∗∗p < 0.01, ∗∗∗p < 0.001, and ∗∗∗∗p < 0.0001.

To examine the roles of specific inclusion of *Gfpt1 exon 9* in the mammalian skeletal muscle, we generated a mouse line deficient for *Gfpt1* exon 9 by CRISPR/Cas9 system (Figures S8A and S8B). In wild-type mice, the ratios of *Gfpt1-L* mRNA were 83% in triceps brachii and 35% in heart (Figure S8C), whereas *Gfpt1-L* mRNA was not detected in either tissue in KO mice (Figure 4A). A marginal difference in predicted molecular weights (2.1 kDa) between Gfpt1-L and Gfpt1-S prevented us from quantifying the ratio of Gfpt1-L and Gfpt1-S at the protein level in wild-type mice. The amount of total *Gfpt1* mRNA was increased 1.3-fold in triceps brachii in KO mice but not in the heart or kidney (Figure 4A). In contrast, total Gfpt1 protein in the triceps brachii in KO mice was 4.5-times higher than that in wild-type mice (Figure 4B). Similarly, total Gfpt1 protein in KO mice was 1.8-times higher in the heart, but was similar in the liver, compared to wild-type mice (Figure S8F). Analysis of the amount of total Gfpt1 protein in primary myoblasts from wild-type and KO mice revealed that the amounts of total Gfpt1 protein were similar between the two primary myoblasts (Figure S8E). However, in primary myotubes, the amount of total Gfpt1 protein in KO myotubes was increased ∼1.5-fold compared to that in wild-type myotubes (Figure S8E). A ∼4.5-fold increase of the GFPT1-S protein with only a 1.3-fold increase of *Gfpt1* mRNA could be accounted for by either higher translation efficiency of GFPT1-S than -L, or higher degradation speed of GFPT1-L than -S. We first predicted the structures of GFPT1-L and -S by AlphaFold2. AlphaFold2 appropriately predicted the dimeric structures of human and mouse GFPT1-S (Figure S9A). In contrast, a segment comprised 18 amino acids encoded by exon 9 in GFPT1-L was predicted to be disorganized or failed to be predicted by AlphaFold2 (loops in Figure S9A). This disorganized loop may make GFPT1-L more unstable than GFPT1-S. We thus analyzed the translation efficiencies and stabilities of GFPT1-S and -L, and found that GFPT1-S and -L were translated at similar levels and were degraded at similar speeds after adding cycloheximide (Figure S9B). Taken together, one of the physiological roles of *Gfpt1* exon 9 is likely to reduce the Gfpt1 protein level in skeletal muscle. However, neither translation efficiency nor protein stability accounted for the effect of *Gfpt1* exon 9.

Since total Gfpt1 protein was elevated in skeletal muscle in KO mice, we examined whether HBP was upregulated in KO mice by quantifying the final product of HBP, UDP-GlcNAc, in skeletal muscle by liquid chromatography/tandem mass spectrometry (LC/MS/MS). As predicted, KO mice had 2.8 times more UDP-HexNAc (UDP-GlcNAc plus UDP-GalNAc) than wild-type mice in the triceps brachii (Figure 4C). Dot blots with RL2 and CTD110.6 antibodies for detecting O-glycosylated proteins (Figure 4D) and both O- and N-glycosylated proteins (Figure 4E), respectively, showed ∼2-times increases of these glycoproteins in skeletal muscle in KO mice. These results suggest that GFPT1-L downregulates O-GlcNAcylation and N-glycosylation in skeletal muscle by attenuating the HBP.

To further analyze the effects of enhanced HBP by lack of *Gfpt1* exon 9, we quantified 116 metabolites in the glycolysis pathway and its downstream pathways, which play a pivotal role in energy metabolism, in skeletal muscle by capillary electrophoresis-mass spectrometry (CE-MS) (Table S2). We observed no significant differences between wild-type and KO mice in adenylate and guanylate energy charges, which are indicators of the intracellular energy status (Figure 4G). Although the NADH/NAD^+^ ratio was not changed in KO mice (Figure 4G), its surrogate markers, the lactate/pyruvate and malate/aspartate ratios, were increased in KO mice by 20% and 50%, respectively (Figure 4H). Metabolites in the glycolysis pathway and the tricarboxylic acid (TCA) cycle tended to be decreased in *Gfpt1* exon 9 KO mice, while statistically significant decrease was observed only in aspartate (∼50% of wild-type mice) (Figure 4F). Asparagine (∼70%), methionine (∼80%), and threonine (∼70%), all of which were downstream to aspartate, were also decreased in KO mice. The urea cycle was also downstream to aspartate, and their metabolites were decreased in KO mice, especially of urea (∼50%) and citrulline (∼70%) (Figure 4F). Thus, in skeletal muscle in KO mice, the activated HBP suppressed glycolysis and reduced aspartate and its downstream metabolites (asparagine, methionine, threonine, and urea cycle), and increased the malate/aspartate ratio. The physiological significance of the metabolomic changes will be addressed in the discussion.

As HBP and its downstream O-GlcNAcylation pathway serve as mediators for nutrient-sensing, and regulate glucose uptake in response to insulin,^51^^,^^52^^,^^53^ we performed an oral glucose tolerance test. The test showed that the blood glucose level was rapidly increased and slowly decreased, with a significantly higher area-under-the-curve (AUC) in KO mice compared to wild-type mice (Figure 4I). Similarly, the insulin tolerance test showed a compromised response to insulin with a significantly higher AUC of blood glucose in KO mice compared to wild-type mice (Figure 4J). Thus, in *Gfpt1* exon 9 KO mice, the compromised response to insulin impaired glucose uptake and increased the blood glucose levels likely through the upregulation of HBP in skeletal muscle.

### Aged *Gfpt1* exon 9 KO mice show impaired motor performances

**Figure 5:**
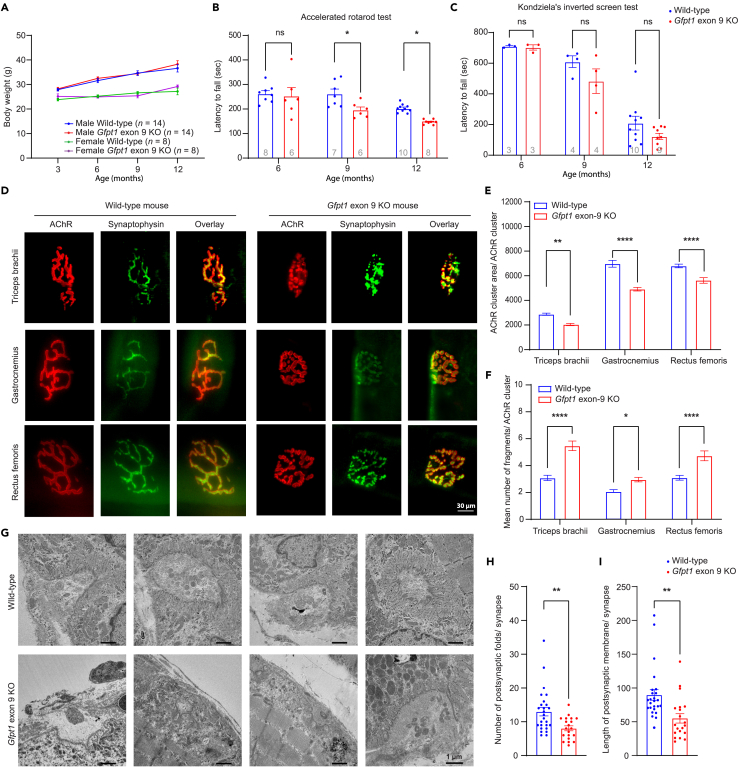
Aged *Gfpt1* exon 9 KO mice show impaired motor performance (A) Temporal profiles of body weights of four indicated groups of mice. The number of mice is indicated in parentheses. (B) Accelerated rotarod test to analyze motor functions of wild-type and *Gfpt1* exon 9 KO mice at indicated ages. The rotarod platform was accelerated from 4 to 40 rpm in 4 min. The number of mice is indicated within the bar. (C) Inverted screen test to analyze motor functions of wild type and *Gfpt1* exon 9 KO mice at indicated ages. The number of mice is indicated within the bar. (D) Representative confocal images of the NMJs of the triceps brachii, gastrocnemius, and rectus femoris muscles stained with anti-synaptophysin antibody (green) and α-bungarotoxin (red) to visualize the nerve terminals and acetylcholine receptors (AChRs), respectively. Scale bar represents 30 μm. (E and F) Quantitative analysis demonstrating AChR cluster area/AChR cluster (E) and mean number of fragments/AChR cluster (F) in wild type and *Gfpt1* exon 9 KO mice (n = 100 NMJs in 4–5 mice each). (G) Representative electron micrographs of the NMJs in the triceps brachii of wild type and *Gfpt1* exon 9 KO mice. Scale bar represents 1 μm. (H and I) Blinded morphometric analyses of the number of postsynaptic folds (H) and the length of postsynaptic membrane normalized to the nerve terminal area (I) in wild type and *Gfpt1* exon 9 KO mice (n = 20–26 NMJs in 4–5 mice each). (A, B, C, E, F, H, and I) Mean and SEM are indicated. Unpaired Student’s *t* test was applied to (C, H, and I). two-way ANOVA with posthoc Tukey test was applied to (A, B, E, and F). ∗p < 0.05, ∗∗p < 0.01, ∗∗∗p < 0.001, and ∗∗∗∗p < 0.0001.

As CMS patients with loss-of-function variants in *GFPT1* demonstrate a limb-girdle pattern of myasthenia,^10^^,^^12^^,^^13^^,^^54^ we analyzed motor functions and the NMJ structures in KO mice. Body weights of KO mice were similar to those of wild-type mice even with aging (Figure 5A). μCT showed that there was no difference in the areas of paravertebral skeletal muscle and fat between wild-type and KO mice at age 12 months (Figure S10A). Similarly, the wet weights of five skeletal muscles and three fat tissues showed no difference between the two groups of mice (Figure S10B). In contrast, motor performance of KO mice evaluated by the rotarod test was similar to wild-type mice up to age 6 months, but was gradually declined from the age 9 months with aging (Figure 5B). Similarly, an inverted screen test showed no difference at age 6 months, but was declined from age 9 months in KO mice, though statistical significance was not observed (Figure 5C). We next asked whether the impaired motor functions in aged KO mice were due to defects at the NMJ, as were observed in *GFPT1*-CMS patients. RNA-seq analysis of skeletal muscles at age 13 months showed that the expression levels of 22 essential genes at the NMJ, which are often defective in CMS, were similar between wild-type and KO mice (Figure S10C). Similarly, no tubular aggregates or centrally located nuclei were observed in myofibers in aged KO mice (Figures S10D and S10E). In contrast, in triceps brachii, gastrocnemius, and rectus femoris muscles of KO mice at ages 12 to 14 months, AChR clusters were fragmented (Figure 5D). Quantitative analyses showed that the AChR cluster areas were significantly reduced (Figure 5E), and the average number of fragmented AChR clusters per motor endplate was significantly increased (Figure 5F), in all the three skeletal muscles in KO mice. The ultrastructures of the NMJ at ages 12 to 14 months showed small nerve terminals with simplified junctional folds (Figure 5G). Quantitative analyses revealed that both the number of junctional folds and the length of the postsynaptic membrane were markedly reduced in KO mice compared to wild-type mice (Figures 5H and 5I). Thus, aged *Gfpt1* exon 9 KO mice showed abnormal NMJ structures as observed in patients with *GFPT1*-CMS.^12^^,^^13^^,^^14^^,^^15^^,^^16^

## Discussion

The striated muscle-specific splicing isoform of *GFPT1* (*GFPT1-L*) was first reported in human and mouse in 2001,^8^^,^^9^ and later in pig in 2010.^55^ However, the splicing regulation of *GFPT1-L* in skeletal muscle remained to be elucidated. The 5′ splice sites are recognized by direct base-pairing with the 5′ end of the U1 small nuclear RNA (snRNA), although recognition is not fully dependent on this pairing.^56^ The 5′ splice site is composed of the last three nucleotides of an exon (positions −3 to −1) and the first six nucleotides of an intron (positions +1 to +6). The consensus sequence of the 5′ splice site is (C/A)AG|gu(a/g)ag(u/g)a, where a vertical line represents an exon-intron boundary.^50^^,^^56^ As observed in other alternatively spliced exons, the 5′ splice site of *GFPT1* exon 9 (GAG|guggga) has a weak splicing signal with non-consensus nucleotides at positions −3, +4, and +6 (Figure 3). Complex interactions between splicing *cis*-elements (enhancers and silencers) and *trans-*acting RBPs determine how and when the alternative splice sites are activated.^57^ Splicing-enhancing and suppressing activities of RBPs are often determined in a binding position-specific manner.^58^ In general, SR proteins enhance and suppress splicing at the 5′ splice site, when they are recruited to an exon and an intron, respectively.^58^ In contrast, hnRNPs enhance and suppress splicing at the 5′ splice site, when they are recruited to an intron and an exon, respectively.^58^ Thus, the position-dependent effects are opposite between SR proteins and hnRNPs. For *GFPT1* exon 9, the splicing-enhancing effect of SRSF1 bound to exon 9 was consistent with the general position-dependency of SR proteins (Figure 3J). Similarly, the splicing-suppressive effect of hnRNP H/F bound to exon 9 was also consistent with the general rule of hnRNPs (Figure 3J). Splicing activation and suppression by SR proteins and hnRNPs are likely to be substantiated in the early spliceosome complex. SRSF1 simultaneously recognizes an exonic splicing enhancer and U1-70K to recruit U1 snRNP at the 5′ splice site, where SRSF1 directly interacts with U1-70K by their respective RNA recognition motif (RRM) domains.^59^ We indeed showed that U1 snRNPs including U1-70K were present in the early spliceosome complex at the 5′ splice site of *GFPT1* exon 9 (Figures 3G–3I). Disruption of the SRSF1-binding site and depletion of SRSF1 protein abrogated, whereas disruption of the hnRNP H/F-binding sites and depletion of hnRNP H/F enhanced, the formation of the early spliceosome complex at the 5′ splice site of *GFPT1* exon 9 (Figures 3G–3I).

Rbfox proteins are neuron- and muscle-specific RBPs that either enhance or suppress splicing. Similar to hnRNPs, the Rbfox binding motif (U)GCAUG activates splicing when located downstream of an exon.^30^^,^^33^^,^^60^^,^^61^^,^^62^^,^^63^^,^^64^^,^^65^ A representative example of splicing enhancement by Rbfox1/2 is observed in exon 16 of *EPB41* encoding the erythrocyte membrane protein band 4.1 in erythroid differentiation, in which binding of Rbfox2 to (U)GCAUG elements in intron 16 recruits U1 snRNP and promotes splicing of exon 16^33^^,^^65^. The authors show that Rbfox2 directly interacts with U1 snRNP-associated proteins, U1C and U1-70K but not U1A. U1C is required for a stable interaction between the pre-mRNA 5′ splice site and U1 snRNP,^66^ although U1C doesn’t directly bind to U1 snRNA. The binding of U1C to the U1 snRNP core domain is mediated by U1-70K through its N-terminal domain.^67^^,^^68^ The N-terminal cysteine/histidine-rich zinc finger-like domain of U1C interacts with the duplex between pre-mRNA and the 5′-end of U1 snRNA, even though it makes no base-specific contacts with pre-mRNA.^69^ The very same domain of U1C is necessary for its interaction with the C-terminal domain of Rbfox2.^65^ U1C thus fine-tunes the affinity to stabilize the binding of U1 snRNA to non-canonical nucleotides at the 5′ splice site. Our findings support the notion that Rbfox1/2 enhances the role of U1C to stabilize the association between the 5′ splice site and U1snRNP (Figure 3).

Rbfox1/2 is preferentially expressed in skeletal and cardiac muscles, as well as various neuronal regions.^70^ In addition, Rbfox1/2 is alternatively spliced in muscles to promote muscle-specific splicing.^70^^,^^71^^,^^72^ SRSF1 and hnRNP H/F are abundantly expressed across different cell and tissue types.^73^ Our data showed that the expressions of Rbfox1/2 were upregulated while those of hnRNP H/F were downregulated during myogenic differentiation (Figures 3A–3D). These results suggest that simultaneous upregulation of Rbfox1/2 and downregulation of hnRNP H/F contribute to the specific inclusion of *GFPT1* exon 9 in skeletal muscles.

The role of GFPT in biosynthesis of glucosamine-6-P from fructose-6-P was first reported by Ghosh et al.^4^ The phylogenetic tree indicates that the first speciation of GFPTs occurred between prokaryotes and eukaryotes, suggesting that prokaryotic and eukaryotic GFPTs can have different functional properties. One such example is that mammalian GFPT is more sensitive to a feedback inhibition by UDP-GlcNAc than prokaryotic GFPT.^74^ Eukaryotic GFPT was split into the invertebrate and vertebrate orthologs, and the vertebrate GFPT was then split into GFPT1 and GFPT2 (Figure S2). Invertebrate GFPTs were more similar to vertebrate GFPT2s than vertebrate GFPT1s (Figure S3), suggesting that GFPT1 was likely to have evolved to exert novel functions in vertebrates. GFPT1 and GFPT2 are ∼76% identical at the amino acid level,^5^ but their tissue expression profiles are different. GFPT1 is highly expressed in the heart, skeletal muscle, placenta, pancreas, and testis, whereas GFPT2 is more expressed in the central nervous system where GFPT1 is less expressed.^5^^,^^7^ *GFPT1* exon 9 and its flanking introns are conserved in mammals (Figure S4), and GFPT1-L is uniquely expressed in skeletal muscle and heart only in mammals (Figure 1D). GFPT1-L has a lower maximum enzymatic activity (∼two-times lower Vmax) and a lower substrate affinity (∼two-times higher K_M_) than GFPT1-S, and is more susceptible to UDP-GlcNAc inhibition (∼five-times lower Ki) than GFPT1-S.^8^^,^^9^

The primary role of UDP-GlcNAc in the HBP is for N- and O-glycosylation of biological molecules, but UDP-GlcNAc also exerts the other essential roles in cell metabolisms. Continuous intravenous administration of glucosamine under an euglycemic hyperinsulinemic clamp increased the UDP-GlcNAc concentration ∼4-fold in skeletal muscle in rat, and downregulated the expression of gene sets in fatty acid oxidation, mitochondrial substrate shuttles, and TCA cycle.^75^ The authors showed that glucosamine significantly decreased whole-body oxygen consumption and energy expenditure. The suppression of the glycolysis pathway and the TCA cycle in *Gfpt1* exon 9 KO mice (Figure 4F) might also be accounted for by a ∼2.8-fold increase of UDP-GlcNAc concentration in skeletal muscle (Figure 4C). In addition to the suppressed glycolysis pathway and TCA cycle, we observed that aspartate and its downstream metabolites were markedly reduced (Figure 4F), which was not addressed in the glucosamine-administered rats.^75^ Cytoplasmic aspartate is converted to malate by accepting electrons from NADH. Malate is then transferred to mitochondria via the malate-aspartate shuttle, and serves as an electron source for the mitochondrial electron transport chain and a carbon source for the TCA cycle. Activation of the malate-aspartate shuttle was thus likely to compensate for the suppressed glycolytic energy production. *Gfpt1* exon 9 KO mice increased UDP-GlcNAc ∼2.8-fold (Figure 4C), but adenylate and guanylate energy charges were preserved (Figure 4G). In accordance with our model, the overexpression of GFPT1 increased UDP-GlcNAc ∼2-fold, but had no effect on ATP production.^76^^,^^77^^,^^78^ In contrast, administration of glucosamine increased UDP-GlcNAc ∼4-fold and reduced the ATP level.^75^ This was likely because a feedback inhibition to the GFPT1 activity was operational in *Gfpt1* exon 9 KO and the overexpression of GFPT1.^75^ In contrast, glucosamine enters the HBP downstream of GFPT1 (Figure 1D), and a feedback inhibition on GFPT1 should have no effect on the production of UDP-GlcNAc. Indeed, glucosamine increased UDP-GlcNAc ∼4-fold, whereas our model and the overexpression of GFPT1^76^^,^^77^^,^^78^ increased it ∼2.8- and ∼2-fold, respectively. Mammals were thus likely have evolved to minimize the production of UDP-GlcNAc by making GFPT1-L for sufficient energy production via glycolysis and TCA cycle in skeletal muscle.

Loss-of-function variants of *GFPT1* cause limb-girdle CMS with tubular aggregates in skeletal muscles, which is characterized by slowly progressive limb-gridle muscle weakness with minor or no involvement of facial, ocular, and bulbar muscles.^10^^,^^12^^,^^13^^,^^54^ *GFPT1*-CMS patients show decreased GFPT1 protein levels in skeletal muscle,^10^^,^^54^ decreased O-GlcNAcylated proteins in skeletal muscles,^10^^,^^12^ and defective neuromuscular signal transmission with decreased AChR.^10^^,^^12^ In accordance with *GFPT1*-CMS patients,^10^^,^^12^^,^^13^^,^^54^ muscle-specific *Gfpt1* KO mice exhibit simplified NMJ with small and fragmented AChR clusters.^17^ In contrast to *GFPT1*-CMS patients^10^^,^^12^^,^^13^^,^^54^ and muscle-specific *Gfpt1* KO mice,^17^ however, *Gfpt1* exon 9 KO mice increased the amounts of GFPT1, as well as of O- and N-glycosylated proteins (Figures 4B–4D). Although the directions of deviations of GFPT1 activities were opposite, simplified NMJs in aged *Gfpt1* exon 9 KO mice were similar to, but were less severe than, those in *GFPT1*-CMS patients^10^^,^^12^^,^^13^^,^^54^ and muscle-specific *Gfpt1* KO mice^17^ (Figure 5D). Similarly, the inverted screen test showed that muscle-specific *Gfpt1* KO mice showed markedly shortened dwell time as early as age 6 weeks,^17^ whereas *Gfpt1* exon 9 KO mice remained normal at age 6 months and tended to decline from age 9 months (Figure 5C). Muscle-specific *Gfpt1* KO mice showed muscle fatigue by *in situ* force measurement of TA muscle at age 3 months.^17^ Although we did not directly measure muscle fatigue *in situ*, the evaluation of the muscle strength and muscle fatigue by the accelerated rotarod test and the inverted screen test showed that abnormal muscle fatigue is unlikely to be present at age 6 months in *Gfpt1* exon 9 KO mice (Figures 5B and 5C). Tubular aggregates and muscle regeneration with centrally located nuclei, which are frequently observed in *GFPT1*-CMS patients^10^^,^^12^^,^^13^^,^^54^ and muscle-specific *Gfpt1* KO mice,^17^ were not observed in *Gfpt1* exon 9 KO mice (Figures S10D and S10E). Additionally, unlike muscle-specific *Gfpt1* KO mice,^17^ AChR subunit genes (*Chrna1*, *Chrnb1*, *Chrnd*, and *Chrne*) were not downregulated and *Musk* was not upregulated in *Gfpt1* exon 9 KO mice (Figure S10C). All these differences point to the notion that *Gfpt1* exon 9 KO mice have milder NMJ and muscle phonotypes than muscle-specific *Gfpt1* KO mice.^17^

In *GFPT1*-CMS, four pathogenic variants affecting *GFPT1* exon 9 have been reported. First, NM_001244710.2:c.686-2A > G at the intron 8/exon 9 boundary activates a cryptic splice site and eliminates the first four nucleotides of exon 9 predicting 56 missense amino acids followed by a stop codon.^12^ Second, NM_001244710.2:c.686dupC predicts NP_001231639.1:p.R230X.^16^ Third, NM_001244710.2:c.706 > T predicts NP_001231639.1: p.K236X.^79^ Fourth, NM_001244710.2:c.719G > A predicts NP_001231639.1: p.W240X.^10^ All the four pathogenic variants generate a stop codon, and O- and N-glycosylated proteins were markedly reduced in a patient with c.686-2A > G.^12^ In contrast to *Gfpt1* exon 9 KO mice, the four patients are likely to have reduced GFPT1 in skeletal muscle. If a pathogenic splicing variant causes skipping of *GFPT1* exon 9, the patient phenotypes are predicted to be similar to those in *Gfpt1* exon 9 KO mice.

In 1996, increased enzymatic activities of GFPT1 were reported in skeletal muscle in patients with NIDDM.^25^ Similarly, in insulin-resistant obese mice, the enzymatic activity of GFPT1 was elevated in the skeletal muscle but not in the liver.^80^ Conversely, overexpression of GFPT1-S in skeletal muscle,^26^ fat,^26^^,^^81^ liver,^77^ and pancreatic β cells,^78^ impaired insulin-mediated glucose update in mice. In accordance with these mouse studies, glucose uptake in response to insulin was impaired in aged *Gfpt1* exon 9 KO mice that had ∼4.5-fold more GFPT1-S in skeletal muscle (Figure 4B). Preservation of the insulin responsiveness is likely to be one of the essential roles of inclusion of *Gfpt1* exon 9 in skeletal muscle. Additionally, type 1 diabetic mice and non-obese diabetic mice show NMJ dysfunctions with reduced acetylcholinesterase (AChE) and AChR fragmentations at the NMJ,^82^^,^^83^^,^^84^ as observed in *Gfpt1* exon 9 KO mice. Thus, the abnormal NMJ phenotypes in *Gfpt1* exon 9 KO mice may involve a pathway similar to that observed in the diabetic mice. In both mouse models, impaired glucose metabolisms may cause the abnormal NMJ phenotypes, although the exact mechanisms still remain unknown. Taken together, GFPT1 is likely to act as a double-edged sword in skeletal muscle, and the finely tuned HBP activity in skeletal muscle is essential for glucose metabolisms, as well as for the formation and maintenance of the NMJ.

### Limitations of the study

We have shown cooperative splicing regulation of *GFPT1* exon 9 by SRSF1, Rbfox1/2, and hnRNP H/F, as well as the roles of GFPT1-L in glucose metabolisms and the NMJ. The primary benefit of acquisition of GFPT1-L in evolution in skeletal muscle in mammals is likely to suppress the HBP for efficient glycolytic energy production in skeletal muscle, as well as the formation and maintenance of the NMJ. However, we could not identify the mechanisms of ∼4.5-fold increase of GFPT1 in the skeletal muscle in *Gfpt1* exon 9 KO mice. Increased UDP-GlcNAc compromised the glucose uptake and the energy production via glycolysis and TCA cycle in skeletal muscle in *Gfpt1* exon 9 KO mice. Similar associations have been repeatedly reported in diabetes mellitus in humans^25^ and mice.^26^^,^^27^^,^^77^^,^^78^^,^^80^^,^^81^ However, the exact underlying mechanisms remain to be elucidated in either study. Additionally, our study was limited to male mice, leaving the comparison to female mice unaddressed. Abnormal NMJ formation is observed in diabetic mice^82^^,^^83^^,^^84^ and in *Gfpt1* exon 9 KO mice. However, the causal relations remain to be solved in both diabetic and our mice. Further studies are required to elucidate the hidden scenarios played by GFPT1-L in skeletal muscle.

## STAR★Methods

### Key resources table

**Table:** 

REAGENT or RESOURCE	SOURCE	IDENTIFIER
**Antibodies**		

anti-SRSF1	Invitrogen	32–4500; RRID: AB_2533079
anti-Rbfox1	Santa Cruz Biotechnology	sc-135476; RRID: AB_2221430
anti-Rbfox2	Bethyl Laboratories	A300-864A; RRID:AB_609476
anti-hnRNP F/H	Santa Cruz Biotechnology	sc-32310; RRID:AB_2248257
anti-U1-70K	Synaptic Systems	203011; RRID:AB_887903
anti-U1A	Thermo Fisher Scientific	PA5-27474; RRID:AB_2544950
anti-U1C	Sigma-Aldrich	SAB4200188; RRID:AB_10640155
anti-GAPDH	Sigma-Aldrich	G9545; RRID:AB_796208
anti-6xHis-tag	Medical & Biological Laboratories	D291-3; RRID:AB_10597733
anti-Flag M2	Sigma-Aldrich	F3165; RRID:AB_259529
anti-U2AF65	Santa Cruz Biotechnology	sc-53942; RRID:AB_831787
anti-GFPT1	Abcam	ab125069; RRID:AB_10975709
anti-RL2	Santa Cruz Biotechnology	sc-59624; RRID:AB_784963
anti-*O*-GlcNAc (CTD110.6)	Cell Signaling Technology	9875; RRID:AB_10950973
anti-β-actin	Santa Cruz Biotechnology	sc-47778; RRID:AB_626632
anti-synaptophysin antibodies	Innovative Research	18–0130; RRID:AB_86671
Alexa 594-conjugated ɑ-bungarotoxin	Invitrogen	B-13423; RRID:AB_2861425
anti-mouse IgG conjugated to horseradish peroxidase	Cell Signaling Technology	7076; RRID:AB_330924
goat anti-rabbit IgG conjugated to horseradish peroxidase	Cell Signaling Technology	7074; RRID:AB_2099233
Alexa 488-conjugated goat anti-rabbit antibody	Thermo Fisher Scientific	A-11034; RRID:AB_2576217

**Bacterial and virus strains**		

*E. coli*	Stratagene	N/A

**Biological samples**		

collagen type-I	Merck	08–115
Human Skeletal Muscle Total RNA	Clontech,	636534

**Chemicals, peptides, and recombinant proteins**		

HeLa nuclear extract	CilBiotech	CC-01-30
Cas9 proteins	New England Biolabs	M0646M
Protease Inhibitor Cocktail	Roche	57035900
phosphatase inhibitor	Roche	59124500
PreScission Protease	GE Healthcare	27-0843-01
Insulin Humulin R	Eli Lilly	N/A
Cycloheximide	FUJIFILM Wako Pure Chemical Co	037–20991

**Critical commercial assays**		

Pierce 660 nm Protein Assay Reagent	Thermo Fisher Scientific	Catalog no. 22660
RNeasy Plus Mini Kit	Qiagen, Tokyo, Japan	Catalog no. 74134

**Experimental models: Cell lines**		

KD3/Hu5	Hashimoto et al., 2006^87^	RCB2366
HEK293 cells	ATCC	CRL-1573
Muscle satellite cells	This paper	Motohashi et al., 2014^88^

**Experimental models: Organisms/strains**		

*Gfpt1* exon 9 KO mice	This paper	Strain: C57BL/6J

**Oligonucleotides**		

single-strand guide RNA	This paper	The target sequence in the single-strand guide RNA (sgRNA, 5′-ATCCACATGGTG GGGATCAC-3′) on *Gfpt1* exon 9 was determined using the CRISPOR website
Primers for PCR, RT-PCR, and qRT-PCR, see Table S1	This paper	N/A
siRNA to *RBFOX1*	This paper	Runfola et al., 2015^89^
siRNA to *RBFOX2*	This paper	Zhou et al., 2007^90^
siRNA to *SRSF1*	This paper	Rahman et al., 2015^91^
siRNA to *HNRNPH1*	Qiagen	SI02654799
siRNA to *HNRNPF*	This paper	Garneau et al., 2005^92^
siRNA to *HNRNPH1 and HNRNPF*	This paper	Garneau et al., 2005^92^
AllStar Negative Control siRNA	Qiagen	1027281

**Recombinant DNA**		

Full-length human *GFPT1-S* cDNA	Open Biosystems	5298729
Full-length human *GFPT1-L* cDNA	This paper	Human GFPT1-L cDNA was amplified using a reverse-transcribed library of Human Skeletal Muscle Total RNA (Clontech, 636534) with KOD plus enzyme
*GFPT1* minigene	This paper	Masuda et al., 2008^44^; Nasrin et al., 2014^45^
SRSF1	This paper	Ahsan et al., 2017^47^; Masuda et al., 2008^44^; Nazim et al., 2017^35^; Rahman et al., 2015^91^
SRSF1-MS2	This paper	Ahsan et al., 2017^47^; Masuda et al., 2008^44^; Nazim et al., 2017^35^; Rahman et al., 2015^91^
HNRNPH1	This paper	Ahsan et al., 2017^47^; Masuda et al., 2008^44^; Nazim et al., 2017^35^; Rahman et al., 2015^91^
HNRNPH1-PP7	This paper	Ahsan et al., 2017^47^; Masuda et al., 2008^44^; Nazim et al., 2017^35^; Rahman et al., 2015^91^
HNRNPF	This paper	Ahsan et al., 2017^47^; Masuda et al., 2008^44^; Nazim et al., 2017^35^; Rahman et al., 2015^91^
HNRNPF-PP7	This paper	Ahsan et al., 2017^47^; Masuda et al., 2008^44^; Nazim et al., 2017^35^; Rahman et al., 2015^91^
RBFOX1	This paper	Human *RBFOX1* cDNA was cloned in the pEFBOS I FLAG/MYC or pGEX-6P-1 vector
Rbfox1-MS2	This paper	MS2 coding sequence was inserted to *RBFOX 1* cDNA construct to make Rbfox1-MS2 fusion proteins
RBFOX2	This paper	Human *RBFOX2* cDNA was cloned in the pEFBOS I FLAG/MYC or pGEX-6P-1 vector
Rbfox2-MS2	This paper	MS2 coding sequence was inserted to *RBFOX 2* cDNA construct to make Rbfox2-MS2 fusion proteins

**Software and algorithms**		

Prism	GraphPad	Version 9
ImageJ	ImageJ	http://imagej.nih.gov/ij/
MetaMorph	MetaMorph	User manual
SkyScan DataViewer	Bruker	User manual
Python script to measure AChR area	This paper	Data S1

**Other**		

Trizol reagent	Thermo Fisher Scientific	15596018
oligo-dT primer	Thermo Fisher Scientific	AM5730G
ReverTra Ace reverse transcriptase	Toyobo	TRT-101
GoTaq polymerase	Promega	M7123
SYBR Premix Ex Taq II	Takara	RR820A
amylose resin beads	New England Biolabs	E8021S
FuGENE 6	Promega	E2691
Lipofectamine RNAiMAX	Thermo Fisher Scientific	13778100
VectaShield mounting medium (DAPI)	Vector Laboratories	H-1200-10
nitrocellulose membrane	BIO-RAD	0.45 μm pore size
Protein G HP spin trap	GE Healthcare	28903134
Glutathione Sepharose beads	GE Healthcare	17-0756-01
protein concentrator columns	Millipore Sigma	UFC501024
Dot Microfiltration apparatus	SCIE-PLAS	DHM48
LightCycler 480 II	Roche	N/A
NEPA 21 electroporator	NEPA GENE	N/A
Rotarod machine	Ugo Basile	N/A
BX53 microscope	Olympus	N/A
FreeStyle Flash Glucometer	Nipro	N/A
LCMS-8060	Shimadzu	N/A
Nexera HPLC system	Shimadzu	N/A
Disposable homogenizer	BioMasher II, Funakoshi	N/A
X-ray-computed microtomography scanner	Bruker	Skyscan 1176
Ultrasonic disruptor	Tomy Digital Biology	UR-21P

### Resource availability

#### Lead contact

Further information and requests for resources and reagents should be directed to and will be fulfilled by the lead contact, Kinji Ohno (ohnok@med.nagoya-u.ac.jp).

#### Materials availability

All developed expression plasmids produced in this study can be made available upon request to the lead contact.

### Experimental model and study participant details

#### Generation of *Gfpt1* exon 9 KO mice by genome editing

All procedures were approved by the Animal Care and Use Committee of Animal Care and Use Committee of the Nagoya University Graduate School of Medicine, and were carried out in accordance with relevant guidelines. The target sequence in the single-strand guide RNA (sgRNA, 5′-ATCCACATGGTGGGGATCAC-3′) on *Gfpt1* exon 9 was determined using the CRISPOR website (http://crispor.tefor.net/). A mixture of 8 μM sgRNA (FASMAC) and 200 ng/μL Cas9 protein (New England Biolabs, Catalog number: M0646M) were incubated at 37°C for 20 min to form a ribonucleoprotein complex. Then the mixture was electroporated into one-cell-stage fertilized eggs obtained from C57BL/6J mice (Japan SLC) using a NEPA 21 electroporator (NEPA GENE). The injected eggs were then transferred into the oviductal ampulla of pseudo-pregnant ICR mice purchased from Charles River Laboratories Japan. All manipulations for generating the mouse line were performed following general procedures in the Animal Facility of Nagoya University Graduate School of Medicine. PCR amplification of the gene sequence around exon 9 was performed with the GoTaq Green Master Mix (Promega, Catalog number: M7123) and primers listed in Table S1. Mutations in *Gfpt1* in offspring were confirmed by Sanger sequencing. Potential off-target sites were predicted by the CRISPOR website (http://crispor.tefor.net/). Eight regions with the highest off-target scores were sequenced, and no mutations were detected. A mouse carried a deletion of 72 nucleotides in intron 8 and exon 9 of *Gfpt1* (chr 6: 87,060,757-87,060,829 according to GRCm38/mm10) (Figure S8A). Homozygous mice were generated and were used as the *Gfpt1* exon 9 KO mice in this communication. Age-matched wild-type C57BL/6J mice (Japan SLC) were used to be compared with the homozygous *Gfpt1* exon 9 KO mice. Although sex differences should exist in the effect of *Gfpt1* exon 9 KO, only male mice were used at the age of 12–14 months throughout our studies.

#### Cell culture and transfection

Immortalized human KD3/Hu5 myoblasts were kindly provided by Dr. Naohiro Hashimoto at the National Center for Geriatrics and Gerontology (NCGG), Japan.^87^ KD3/Hu5 myoblasts were grown on collagen type-I (rat tail, Merck, 08–115) coated dishes with high-glucose (4.5 g/mL) DMEM (hDMEM) medium containing 20% fetal bovine serum and 2% Ultroser G serum substitute (Biosepra, PALL, 292050), as previously described.^45^ To induce myogenic differentiation of KD3/Hu5 myoblasts, the culture medium of confluent cells was switched to hDMEM medium supplemented with 2% fetal calf serum and ITS containing 10 μg/mL insulin, 5 μg/mL bovine holo-transferrin, and 10 nM selenite (Thermo Fisher Scientific, 41400045). Mammalian expression plasmids, indicated below, were transfected by FuGENE 6 (Promega, E2691), whereas siRNA duplexes were transfected by Lipofectamine RNAiMAX transfection reagent (Thermo Fisher Scientific), both according to the manufacturer’s instructions.

Muscle satellite cells were isolated from 1 or 2 young adult mice (6–8 weeks) as previously described.^88^ Primary myoblasts were grown on Matrigel-coated (Sigma-Aldrich, CLS356231) dish in DMEM medium containing 20% FBS. To induce myogenic differentiation, the culture medium of confluent cells was switched to DMEM containing 5% horse serum.

HEK293 cells were cultured in Dulbecco’s Modified Eagle’s medium (DMEM; Gibco) supplement with 10% fetal bovine serum (FBS). HEK293 cells were transfected with 1 μg of human *GFPT1-S* and *GFPT1-L* plasmids using FuGENE 6 Transfection Reagent (Promega) according to the manufacturer’s recommendations. At 24 h after transfection, HEK293 cells were treated with 20 μM cycloheximide (FUJIFILM Wako Pure Chemical Co, 037–20991), and the cells were harvested at the indicated time point.

### Method details

#### Calculation of percent spliced-in (PSI) of Gfpt1 genes

To analyze PSI values of *GFPT1* exon 9 in nine vertebrates, we downloaded eight publicly available RNA-seq datasets with the accession numbers of GSE41637, GSE77020, and ERP015966. Mapping was performed using STAR (v 2.7.9a)^93^ with default parameters against the genome for *Macaca mulatta* (Mmul_10), *Bos taurus* (ARS-UCD1.2), *Rattus norvegicus* (mRatBN7.2), or *Mus musculus* (GRCm39). We counted the junction reads bridging exons 8–9 and exons 9–10 as the reads supporting exon inclusion. In contrast, we counted the junction reads bridging exons 8–10 as the reads supporting exon exclusion. We calculated the PSI of an exon in each RNA-seq dataset according to the following equation:

PSI = (0.5 × inclusion-supporting reads)/(exclusion-supporting reads + 0.5 × inclusion-supporting reads).

#### Construction of GFPT1 expression vectors and minigene

Full-length human *GFPT1-S* cDNA (Open Biosystems, 5298729) was first cloned into BamHI and EcoRI sites of the pcDNA3.1 (+) vector (Thermo Fisher Scientific). Human *GFPT1-L* cDNA was amplified using a reverse-transcribed library of Human Skeletal Muscle Total RNA (Clontech, 636534) with KOD plus enzyme and RT-PCR primers: 5′-CCGGATCCGGCATCATGTGTGGTATATTTGC-3’ (BamHI-ATG) and 5′-GGGAATTCTATTCCTCACTCTACAGTCACAG-3’ (Stop-EcoRI), where restriction sites are underlined. The PCR product was inserted in pcDNA3.1(+) after digestion with BamHI and EcoRI. Presence of exon 9, as well as absence of artifacts, were confirmed by sequencing the entire insert. Then, human *GFPT1-S* and *GFPT1-L* cDNAs were transferred to the BamHI and NotI sites of the pcDNA3.1D/V5-His TOPO mammalian expression vector (Thermo Fisher Scientific) containing the six-histidine tag (6xHis) and the V5 tag at the C-terminus.

We constructed human *GFPT1* minigene by inserting exon 9 and its flanking intronic regions (120 nucleotides of intron 8 and 100 nucleotides of intron 9; genome coordinates from 69,581,290 to 69,581,564 on chromosome 2 according to GRCh37/hg19 in the modified exon-trapping vector, pSPL3.^44^^,^^45^ PCR primers carried NotI and PacI sites to be inserted to pSPL3. Artificial mutations were introduced in the minigene using the QuikChange Site-Directed Mutagenesis Kit (Agilent, 200518).

Constructions of plasmids carrying human *SRSF1*, *SRSF1*-MS2, *HNRNPH1*, *HNRNPH1*-PP7, *HNRNPF*, and *HNRNPF*-PP7 cDNAs were previously reported.^35^^,^^44^^,^^47^^,^^91^ Human *RBFOX1* and *RBFOX2* cDNAs were cloned in the pEFBOS I FLAG/MYC vector. We inserted the MS2 coding sequence to *RBFOX1* and *RBFOX2* cDNA constructs to make Rbfox1-MS2 and Rbfox2-MS2 fusion proteins, respectively. To make recombinant GST-tagged Rbfox1 and Rbfox2 proteins, we cloned human *RBFOX1* and *RBFOX2* cDNAs in pGEX-6P-1 vector.

#### RT-PCR and quantitative RT-PCR (qRT-PCR)

Total RNA was extracted from cultured cells at 48 h after transfection using Trizol reagent (Thermo Fisher Scientific) followed by DNase I (Qiagen) treatment. Total RNA was extracted from triceps brachii muscle of wild-type and *Gfpt1* exon 9 KO mice using Trizol reagent followed by the RNeasy Plus Mini Kit (Qiagen, Tokyo, Japan) according to the manufacturer’s instructions. We synthesized cDNAs with an oligo-dT primer (Thermo Fisher Scientific) and ReverTra Ace reverse transcriptase (Toyobo). PCR was performed with GoTaq polymerase (Promega) using primers listed in Table S1 qRT-PCR was performed using SYBR Premix Ex Taq II (Takara) and LightCycler 480 II (Roche) to quantify endogenous human *SRSF1*, *RBFOX1*, *RBFOX2*, *HNRNPH1*, and *HNRNPF* transcripts using primers shown in Table S1. Values were normalized with endogenous human *GAPDH*.

#### siRNA-mediated gene knockdown

Human *RBFOX1* and *RBFOX2* were knocked down by previously reported siRNAs with the sequences of 5′-CCCAGACACAACCUUCUGAAA-3′ and 5′-CCUGGCUAUUGCAAUAUUU-3′, respectively.^89^^,^^90^ Human *SRSF1* was knocked down by siRNA with the sequence of 5′-CCAAGGACAUUGAGGACGUUU-3’.^91^ Human *HNRNPH1* was knocked down with HP Validated siRNA (SI02654799, Qiagen). Human *HNRNPF* was knocked down by siRNA with the sequence of 5′-GCGACCGAGAACGACAUUU-3’.^92^ Human *HNRNPH1* and *HNRNPF* were knocked down together by siRNA against a shared sequence of 5′-GGAAGAAAUUGUUCAGUUC-3’.^92^ We used AllStar Negative Control siRNA (1027281, Qiagen) as a control.

#### Tethered function assay of SRSF1, Rbfox1/2, and hnRNP H/F

We performed artificial tethering of SRSF1, Rbfox1, and Rbfox2 by co-transfection of a reporter minigene carrying an MS2-hairpin loop (5′-ACATGAGGATCACCCATGT-3′) and an effector construct carrying either SRSF1, Rbfox1 or Rbfox2 fused to the bacteriophage MS2 coat protein. We performed artificial tethering of hnRNP H and hnRNP F by co-transfection of a reporter minigene carrying a PP7-hairpin loop (5′-GGCACAGAAGATATGGCTTCGTGCC-3′) and an effector construct carrying either hnRNP H or hnRNP F fused to the bacteriophage PP7 coat protein. We introduced MS2 and PP7 hairpin sequences in the minigene by replacing the native *cis*-element motifs in the *GFPT1* gene using QuikChange Site-Directed Mutagenesis Kit (Agilent).

#### MS2 affinity isolation of early spliceosomal E-complex

We performed MS2-affinity isolation of the early spliceosomal complex as previously described^46^^,^^91^ with minor modifications. One pmol of the RNA probe (Wt-3xMS2, SRSF1_BSmut-3xMS2, Rbfox_BSmut-3xMS2, or hnRNP_H/F_BSmut-3xMS2) was incubated with a 20-fold molar excess of the MS2-MBP fusion protein. Fifty μL of HeLa nuclear extract (CilBiotech) was pre-incubated with 10 μL of amylose resin beads (New England Biolabs) overnight at 4°C. Recombinant Rbfox1 and Rbfox2 proteins (10 ng/μL each) were added to HeLa nuclear extracts depending on experimental conditions. Pretreated HeLa nuclear extract was incubated with the mixture of the RNA probe and the MS2-MBP fusion protein at 37°C for 30 min, followed by the addition of amylose resin beads (50 μL) and incubation at 4°C for 30 min with gentle rotation. The amylose resin was washed four times with the wash buffer [20 mM HEPES (pH 8.0), 150 mM KCl, and 0.05% Triton X-100], and the bound proteins were eluted with 10 mM maltose solution and subjected to immunoblot analyses.

#### Depletion of SRSF1 and hnRNP H/F from HeLa nuclear extract

SRSF1 and hnRNP H/F were depleted from HeLa nuclear extract (CilBiotech, CC-01-30) with anti-SRSF1 (Invitrogen, 32–4500) and anti-hnRNP F/H (Santa Cruz Biotechnology, sc-32310) antibodies, respectively, using Protein G HP spin trap (GE Healthcare, 28903134) following the manufacturer’s instructions.

#### Expression and purification of recombinant proteins

Glutathione S-transferase (GST)-tagged Rbfox1 and Rbfox2 recombinant proteins were affinity-purified by coupling to Glutathione Sepharose beads (GE Healthcare, 17-0756-01) according to the manufacturer’s instructions. Recombinant GST-Rbfox1 and GST-Rbfox2 were cleaved with PreScission Protease (GE Healthcare, 27-0843-01) to remove the GST-tag. Purified recombinant Rbfox1 and Rbfox2 proteins were concentrated using protein concentrator columns (Amicon Ultra-0.5 mL Centrifugal Filter Unit, Millipore Sigma, UFC501024) following the manufacturer’s instructions.

#### Immunoblotting of cultured cells and muscle tissues

KD3/Hu5 cells were washed in PBS with 1×Protease Inhibitor Cocktail (Thermo Fisher Scientific), followed by centrifugation at 2,000 ×*g* for 5 min. The pellets were resuspended in the lysis buffer [10 mM HEPES-NaOH (pH 7.8), 0.1 mM EDTA, 10 mM KCl, 1 mM DTT, 0.5 mM PMSF, 0.1% NP-40, 1× Protease Inhibitor Cocktail] and kept on ice for 10 min. Following sonication, samples were centrifuged at 20,000 ×*g* for 10 min to remove cell debris, and supernatants were collected as total cell lysates for immunoblotting.

Muscle, heart, and liver tissues were homogenized in GFPT buffer (50 mM KH_2_PO_4_, 10 mM EDTA, 5 mM reduced L-glutathione, 12 mM D-glucose-6-phosphate Na_2_, 10 mM PMSF, 1 mM pepstatin A, and 10 mM phosphatase inhibitor [pH 7.6])^10^ using a disposable homogenizer (BioMasher II, Funakoshi). Samples were mixed for 30 min on the rotator at 4°C, sonicated 4 times for 10 s using an ultrasonic disruptor (UR-21P, Tomy Digital Biology), and centrifuged at 17,900 ×*g* for 20 min. The supernatant was collected, and protein concentration was measured with the Pierce 660 nm Protein Assay Reagent (22660, Thermo Fisher Scientific) according to the manufacturer’s instructions. The supernatant was incubated with an equal volume of 2×SDS Sample Buffer [0.125 M Tris-HCl (pH 6.8), 4% (w/v) SDS, 20% (v/v) glycerol, and 0.01% (w/v) bromophenol blue] at 95°C for 5 min, and subjected to immunoblot analyses. Coomassie gel staining was performed to normalize the loading amount in Western blots.

Mouse primary myoblasts and HEK293 cells were washed in PBS with 1×Protease Inhibitor Cocktail (Thermo Fisher Scientific), followed by centrifugation at 2,000 ×g for 5 min. The pellets were resuspended in the lysis buffer (50 mM HEPES, 150 mM NaCl, 1.5 mM MgCl2, 1 mM EGTA, 100 mM NaF, 10 mM sodium pyrophosphate, 10% glycerol, 1% Triton X-100, 10 mM PMSF, 1 mM pepstatin A, and 10 mM phosphatase inhibitor [pH 7.0]) and mixed for 1 h on the rotator at 4°C. Samples were centrifuged at 20,000 ×g for 10 min to remove cell debris, and supernatants were collected as total cell lysates for immunoblotting.

Proteins were separated by electrophoresis on a 7.5% SDS–polyacrylamide gel in Tris-Glycine and transferred onto a polyvinylidene difluoride membrane (PVDF, Immobilon-P, 0.45 or 0.2 μm, Merck Millipore). The membrane was washed in Tris-buffered saline containing 0.05% Tween 20 (TBS-T) and blocked for 1 h at room temperature in TBS-T with 3% bovine serum albumin (BSA) or 5% Non-Fat Dry Milk (NFDM). The membrane was incubated overnight at 4°C with primary antibody. The membranes were washed 3 times for 10 min with TBS-T and incubated with a secondary antibody for 1 h at room temperature. The blots were visualized using Amersham ECL Western blotting detection reagents and quantified with ImageJ software.

#### Antibodies

Antibodies used for immunoblotting were anti-SRSF1 (1:1000, Invitrogen, 32–4500), anti-Rbfox1 (1:100, N-14, Santa Cruz Biotechnology, sc-135476), anti-Rbfox2 (1:2000, Bethyl Laboratories, A300-864A), anti-hnRNP F/H (1:500, Santa Cruz Biotechnology, sc-32310), anti-U1-70K (1:1000, Synaptic Systems, 203011), anti-U1A (1:1000, Thermo Fisher Scientific, PA5-27474), anti-U1C (1:1000, Sigma-Aldrich, SAB4200188), anti-GAPDH (1:2500, Sigma-Aldrich, G9545), anti-6xHis-tag (1:2500, Medical & Biological Laboratories, D291-3), anti-Flag M2 (1:1000, Sigma-Aldrich, F3165), anti-U2AF65 (1:400, MC3, Santa Cruz Biotechnology, sc-53942), anti-GFPT1 (1:500, Abcam, ab125069), anti-RL2 (1:800, Santa Cruz Biotechnology, sc-59624), anti-*O*-GlcNAc (CTD110.6) (1:2000, Cell Signaling Technology, 9875), anti-β-actin (1:1000, Santa Cruz Biotechnology, sc-47778), and anti-synaptophysin antibodies (1:50, Innovative Research, 18–0130). The secondary antibodies were anti-mouse IgG (1:2000, Cell Signaling Technology, 7076) conjugated to horseradish peroxidase, goat anti-rabbit IgG (1:2000, Cell Signaling Technology, 7074) conjugated to horseradish peroxidase, and Alexa 488-conjugated goat anti-rabbit antibody (1:1000, Thermo Fisher Scientific, A-11034). AChRs were visualized by Alexa 594-conjugated ɑ-bungarotoxin (1:1000, Invitrogen, B-13423).

#### LC/MS analysis for quantification of HexNAc

Muscle tissues (20–40 mg) were weighed and quickly frozen in liquid nitrogen. Cellular extracts for nucleotide sugar analysis were prepared as reported previously.^94^ Hydrophilic interaction liquid chromatography and electrospray tandem mass spectrometry (HILIC-ESI-MS/MS) was performed on an LCMS-8060 (Shimadzu) coupled with a Nexera HPLC system (Shimadzu). Chromatography was performed on a BEH-amido column (2.1 mm i.d. x 150 mm, 3 μm; Waters).^95^^,^^96^ Analysis of nucleotide sugars was conducted in the multiple reaction monitoring mode using specific precursor ion [M-H]^-^ and product ions pairs as follows: UDP-HexNAc, *m/z* 606.1→384.7. The nucleotide sugar levels were normalized as pmol/mg tissues.

#### Immunodot blot assay of O-GlcNAc of skeletal muscle

The Dot Microfiltration apparatus (SCIE-PLAS, DHM48) was used to analyze the O-GlcNAc level in the lysates of skeletal muscle. A nitrocellulose membrane (0.45 μm pore size, BIO-RAD) was placed in the apparatus, and protein extracts (0.8 mg in 2 μL) were transferred to the membranes by water vacuum for 20 min. The membrane was incubated with blocking solution containing 3% BSA in Tris-buffered saline with 0.01% Tween 20 (TBS-T) for 60 min at room temperature. The membrane was incubated overnight at 4°C with a primary antibody. The membrane was washed 3 times for 10 min with TBS-T and incubated with a secondary antibody for 1 h at room temperature. The blots were visualized using Amersham ECL Western blotting detection reagents and quantified with the ImageJ software.

#### Metabolomic analysis of skeletal muscle in Gfpt1 exon 9 KO

After 20 h fasting, the triceps brachii muscles were isolated from wild-type and *Gfpt1* exon 9 KO mice at age 13 months (n = 3 mice each). Muscle tissues (20–40 mg) were weighed and quickly frozen in liquid nitrogen. The capillary electrophoresis time-of-flight mass spectrometry (CE-TOFMS)–based metabolome analysis was performed at Human Metabolome Technologies. Metabolites were identified by comparing the migration time and m/z ratio with authentic standards and quantified by comparing their peak areas with those of standards.

Adenylate and guanylate energy charges were calculated by the following formulas using the metabolomic data shown in Table S2:

Adenylate energy charge $=\frac{\left[ATP\right]+0.5x\left[ADP\right]}{\left[ATP\right]+\left[ADP\right]+\left[AMP\right]}$

Guanylate energy charge $=\frac{\left[GTP\right]+0.5x\left[GDP\right]}{\left[GTP\right]+\left[GDP\right]+\left[GMP\right]}$.

#### Oral glucose tolerance test and insulin tolerance test

An oral glucose tolerance test was performed on wild-type (n = 4) and *Gfpt1* exon 9 KO (n = 5) mice at age 12 months. After 12 h fasting, glucose solution (1.0 g/10 mL/kg) was orally administered, and glucose level was measured in the tail vein using a FreeStyle Flash Glucometer (Nipro) at the indicated time points. An insulin tolerance test was performed on wild-type (n = 6) and *Gfpt1* exon 9 KO (n = 5) mice at age 12 months. After 4 h fasting, insulin (0.75 U/kg, Humulin R, Eli Lilly) was intraperitoneally injected, and glucose level was measured in the tail vein using the Glucometer at the indicated time points.

#### Micro-computed tomography (μCT) imaging

At ages 13–14 months, wild-type and *Gfpt1* exon 9 KO mice (n = 5 mice each) were sacrificed and transverse μCT images were taken with a Skyscan 1176 X-ray-computed microtomography scanner (Bruker) and a 0.5-mm aluminum filter. We reconstructed three axial slices at the L4/5 disc level, the femoral lesser trochanter level, and the maximum calf-circumference level with SkyScan DataViewer (Bruker). Paravertebral muscles and fat tissues were manually identified and were quantified using the ImageJ program (http://imagej.nih.gov/ij/).

#### Wet weight of skeletal muscles and white adipose tissues

The biceps brachii, triceps brachii, quadriceps femoris, gastrocnemius, and soleus muscles were dissected from 12-month-old wild-type and *Gfpt1* exon 9 KO mice (n = 3 mice each), and weighed at 0.1 mg resolution (Sartorius, CPA64). Additionally, subcutaneous inguinal, perigonadal, and mesenteric white adipose tissues were dissected, and weighed at 0.1 mg resolution (Sartorius, CPA64).

#### Evaluation of motor performances

Two different tests were performed to evaluate motor functions: the accelerated rotarod test and the modified Kondziela’s inverted screen test.^97^ First, wild-type and *Gfpt1* exon 9 KO mice were subjected to the accelerated rotarod test (Ugo Basile) at ages 6, 9, and 12 months. Mice were first trained on the rotating rod that accelerated from 4 to 40 rpm in 4 min for two consecutive days to accommodate the task. On the third day, mice were placed on the rod, and the time stayed on the rod was recorded. The experiment was consecutively repeated four times, and mice were allowed to rest for 12 min between individual tasks. The observer was blinded to the genetic identity of the examined mice. Second, wild-type and *Gfpt1* exon 9 KO mice were subjected to a modified version of Kondziela’s inverted screen test^97^ at ages 6, 9, and 12 months. We made an inverted screen apparatus of 1.2 cm square wire mesh made of 1 mm wire according to the previous report.^97^ Mice were placed at the center of a top of a wire mesh, and the screen was immediately rotated to an inverted position 30 cm above a flat surface covered with soft paper chips. Mice were first trained four consecutive times in a day. The time stayed under the inverted screen was recorded. The mouse was observed for up to 720 s, and 720 s was recorded if the mouse did not fall from the screen. The numbers of mice ranged from 3 to 10, and the exact numbers are indicated in Figures 5B and 5C.

#### Immunofluorescence microscopy

The triceps brachii, gastrocnemius, and rectus femoris muscles were isolated from wild-type and *Gfpt1* exon 9 KO mice to exclude gender differences (4–5 male and female) at the age of 12 months. Then muscles were fixed in 4% paraformaldehyde (PFA) for 1 h. Muscles were teased into muscle fibers in phosphate-buffered saline (PBS) and were incubated for 15 min in PBS with 100 mM glycine at room temperature to inactivate any remaining free aldehyde. After washing, the teased muscle fibers were incubated in a blocking buffer containing 2% BSA, 5% goat serum, and 0.5% Triton X-100 in PBS for 1 h at room temperature, followed by overnight incubation at 4°C with anti-synaptophysin antibody (1:50, Thermo Fisher Scientific, 18–0130). After removing primary antibody and repeated washes with PBS containing 1% Triton X-100 (PBS-T), the fibers were incubated with Alexa 488-conjugated goat anti-rabbit secondary antibody (1:1000, Thermo Fisher Scientific, A-11034) and Alexa 594-conjugated ɑ-bungarotoxin (1:1000, Thermo Fisher Scientific, B-13423) for 1 h. Residual antibodies and ɑ-bungarotoxin were removed by repeated washes in PBS-T. Finally, the fibers were placed on a glass slide and cover-slipped with VectaShield mounting medium containing 1.5 μg/mL 4′, 6- diamidino-2-phenylindole (DAPI) (Vector Laboratories). The NMJs were visualized using a BX53 microscope (Olympus) and MetaMorph software. To quantify the area of AChR clusters, we wrote a Python script to measure the total area of Alexa Fluor 594 signals (the red fluorescent pixels) with intensity above an arbitrarily defined threshold (Data S1). The number of AChR fragmentations was quantified by two blinded observers, and the mean was taken.

#### Electron and light microscopies

The triceps brachii muscles of wild-type and *Gfpt1* exon 9 KO mice were analyzed at ages 12 to 14 months (n = 4–5 mice each). The triceps brachii muscle was isolated and fixed in 4% PFA overnight at 4°C. Cholinesterase was partially stained using the Ellman method, and the cholinesterase-positive regions were isolated and minced into ∼1-mm^3^ blocks. The excised blocks were fixed with 2% glutaraldehyde for 2 h, treated with 1% OsO_4_, dehydrated in ethanol, and embedded in Epon 812 (TAAB). Ultrathin sections of 60–70 nm were made and stained with uranyl acetate and lead citrate. The NMJs were identified by inspecting the whole ultrathin sections using a JEM-1400PLUS Transmission Electron Microscope. The number of postsynaptic folds was counted for each nerve terminal, and the length of the postsynaptic membrane was quantified using the ImageJ program (http://imagej.nih.gov/ij/).

The triceps brachii muscles were cut into 10-μm sections using a cryostat (CM3050S, Leica), and were stained with hematoxylin and eosin according to the standard procedures. Slides were analyzed using an IX71 microscope (Olympus).

#### High-throughput RNA sequencing (RNA-seq)

Total RNA was harvested from the triceps brachii muscle of wild-type and *Gfpt1* exon 9 KO mice at age 13 months (n = 3 mice each) using Trizol Reagent (Thermo Fisher Scientific) followed by RNA isolation with the RNeasy Plus Mini Kit (Qiagen, Tokyo, Japan) according to the manufacturer’s instructions. The quality of the RNA samples was examined by an Agilent TapeStation, and the following thresholds were applied: quantity >50 ng, concentration >1 ng/mL, no contamination of DNA, and RIN >8.5. RNA-seq was performed at Macrogen, where a sequencing library was prepared using the TruSeq Stranded mRNA kit (Illumina). The library was read on Illumina NovaSeq 6000 (150 bp paired-end reads). Raw reads were trimmed by Trimmomatic v0.39.^98^ Transcripts per million (TPM) of each gene was calculated by Salmon v1.5.0^99^ with default parameters, and then differential gene expressions between wild-type and *Gfpt1* exon 9 KO mice were analyzed by DESeq2 v1.32.0.^100^ The RNA-seq data were deposited in the DDBJ Read Archive (DRA) with the accession numbers of DRA017016 for both wild-type and GSE161601 *Gfpt1* exon 9 KO mice.

### Quantification and statistical analysis

Data are shown as Mean and SEM or mean and SD as indicated in each figure legend. Individual biological replicates have been indicated in each figure legend and scatter-and-bar plots when it is applicable. Statistical analyses were carried out by GraphPad Prism (version 9.3.1) using unpaired Student’s *t*-test, Welch’s *t*-test, multiple unpaired *t*-tests, or one- or two-way ANOVA with posthoc Tukey test as indicated in each figure legend. p values less than 0.05 were considered significant.

## Data Availability

• The RNA-seq data were deposited in the DDBJ Read Archive (DRA) with the accession numbers of DRA017016 for both wild-type and GSE161601 Gfpt1 exon 9 KO mice. Original data supporting the findings of this study are publicly available at Mendeley Data as of the date of publication (https://doi.org/10.17632/d3ksrk8v5n.1). • All original code is available in this paper’s supplemental information. • Any additional information required to reanalyze the data reported in this work is available from the lead contact upon request.

## Supporting citations

The following reference appears in the Supplemental information: ^85^^,^^86^.
