# Supplementary material for: Splicing regulation of GFPT1 muscle-specific isoform and its roles in glucose metabolisms and neuromuscular junction
Source: iScience. 2023 Aug 26;26(10):107746. doi: 10.1016/j.isci.2023.107746 (PMC10514471; doi:10.1016/j.isci.2023.107746)
Supplement: Document S1. Figures S1–S10, Tables S1, S2, and Data S1 [file mmc1.pdf]

iScience, Volume *26*

Supplemental information

Splicing regulation of GFPT1 muscle-specific isoform and its roles in glucose metabolisms and neuromuscular junction

Paniz Farshadyeganeh, Mohammad Nazim, Ruchen Zhang, Bisei Ohkawara, Kazuki Nakajima, Mohammad Alinoor Rahman, Farhana Nasrin, Mikako Ito, Jun-ichi Takeda, Kenji Ohe, Yuki Miyasaka, Tamio Ohno, Akio Masuda, and Kinji Ohno

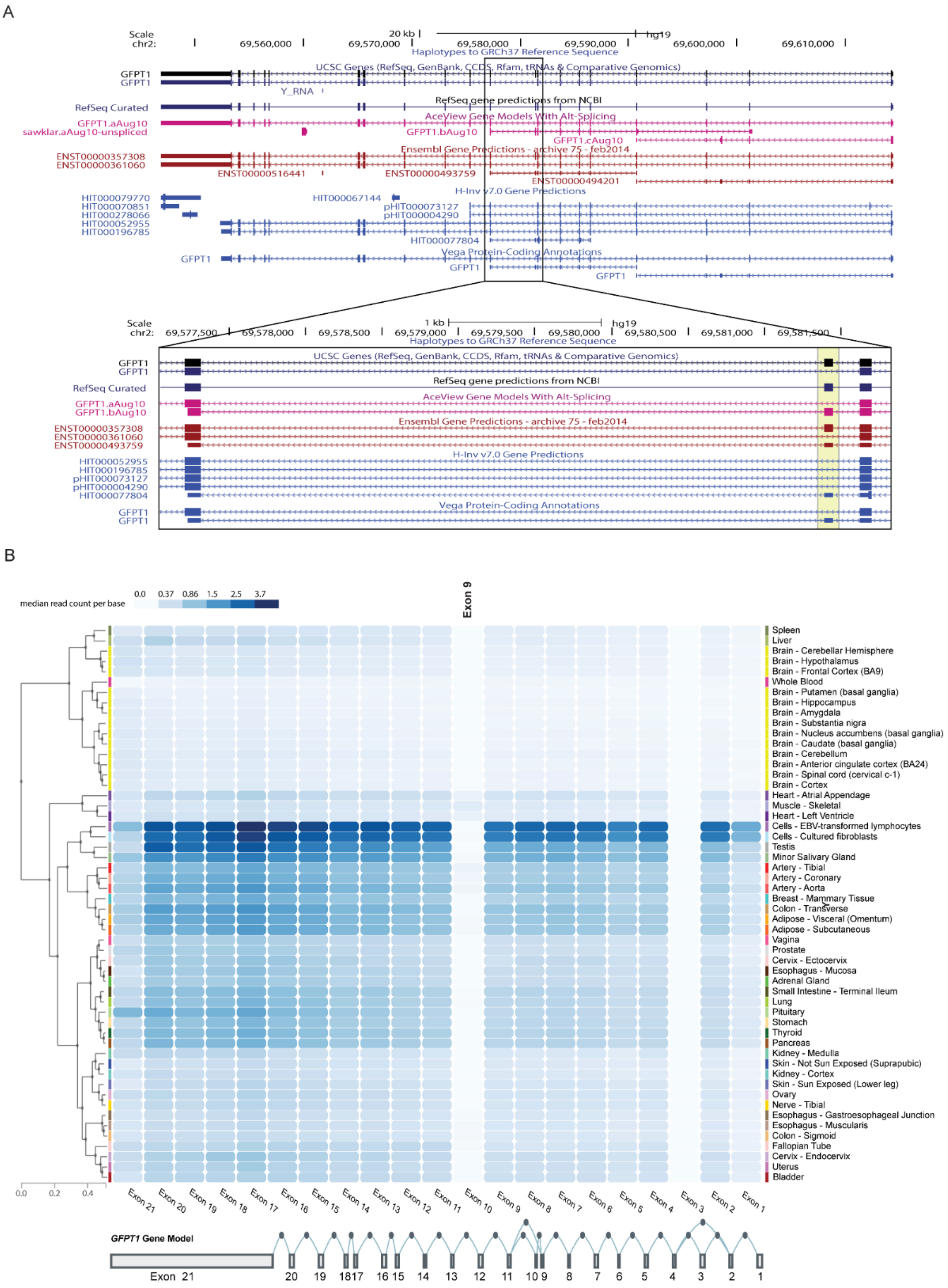

Supplementary Figure S1, related to Figure 1AB

Supplementary Figure S1. Alternative splicing of human *GFPT1* exon 9, related to Figure 1AB.
(A) Alternative transcripts of human *GFPT1* gene annotated in the UCSC Gene, RefSeq, AceView, Ensembl, H-Inv, and Vega databases on the human genome GRCh37/hg19. Exons are shown by boxes, and introns are shown by lines with multiple small arrowheads showing the direction of transcription. Human *GFPT1* is encoded on the opposite strand. Alternatively spliced *GFPT1* exon 9 is shaded yellow in a magnified field. **(B)** Heatmap of the expression levels of each exon in human *GFPT1* in 53 human tissues by the Genotype-Tissue Expression (GTEx) project (https://www.gtexportal.org/home/gene/GFPT1). Exons are indicated in the horizontal axis, and tissues are indicated in the right vertical axis. The median read count per base, which represents the expression level of each exon, is indicated in color code. *GFPT1* exon 9 in this report corresponds to exon 10 in GTEx. Splicing of *GFPT1* is schematically shown at the bottom.

Supplementary Figure S1, related to Figure 1AB

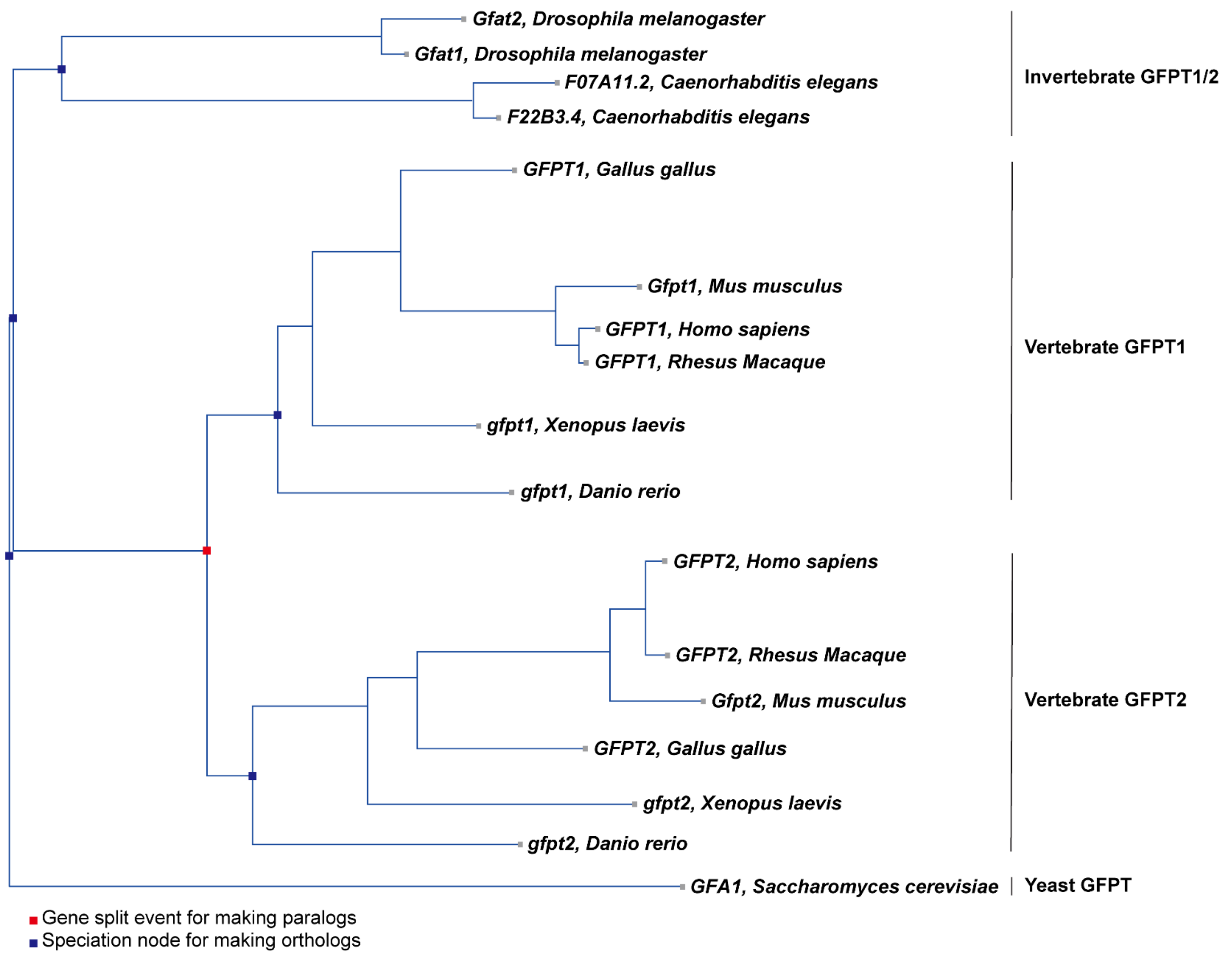

Supplementary Figure S2. Phylogenetic tree of the *GFPT* gene family in six vertebrates, two invertebrates, and yeast, related to Figure 1E. Phylogenetic tree was obtained from the Gene Tree module of the Ensembl database, and was redrawn.

Supplementary Figure S2, related to Figure 1E

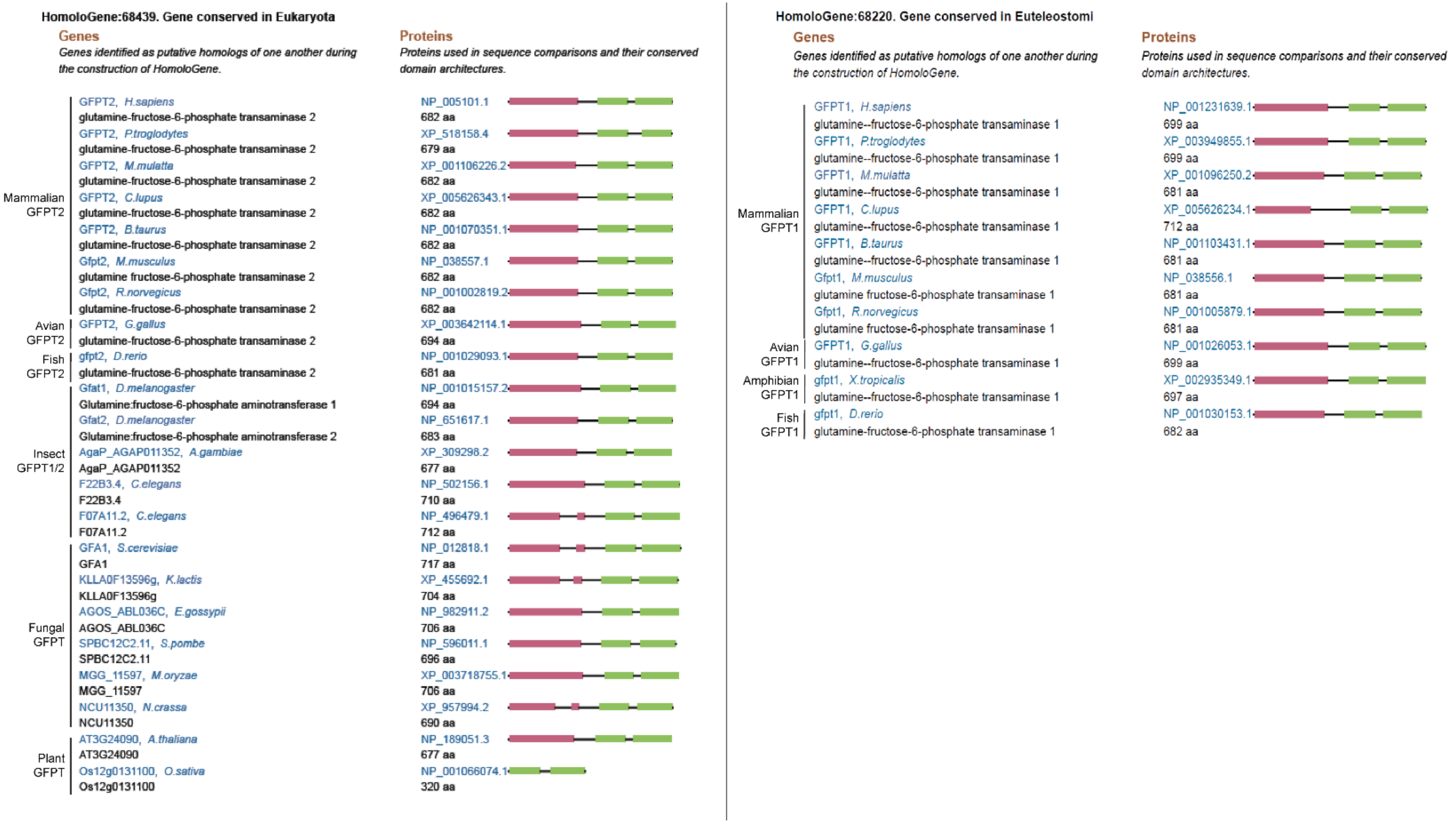

Supplementary Figure S3. Conservation of the domains in GFPT1 and GFPT2 in eukaryotes by the HomoloGene project at the NCBI, related to Figure 1E. Homologs of mammalian GFPT1 and GFPT2 are indicated on the right and left panels, respectively.

Supplementary Figure S3, related to Figure 1E

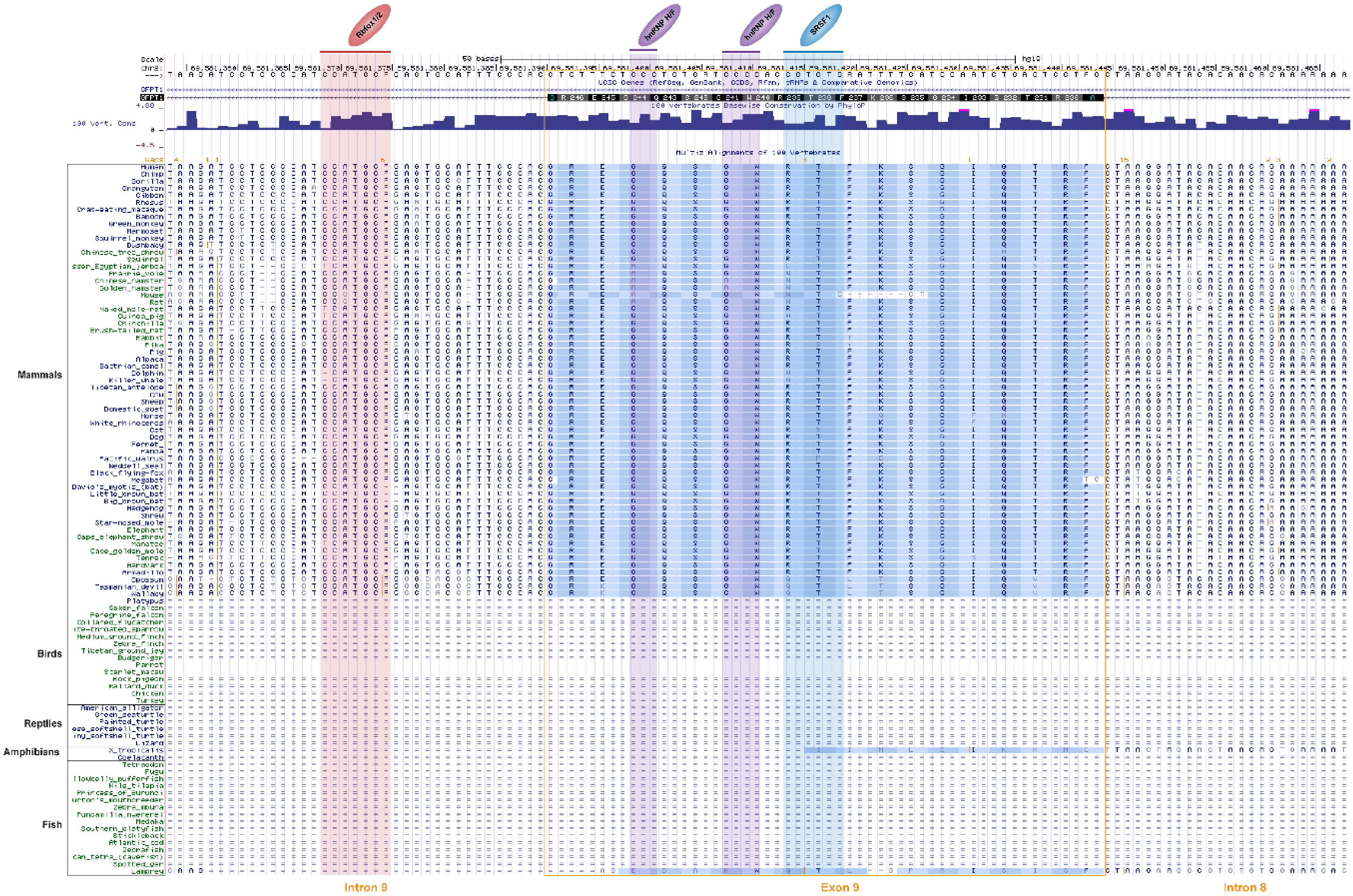

Supplementary Figure S4, related to Figure 1E

Supplementary Figure S4. Screenshot of the UCSC Genome Browser showing the conservation of *GFPT1* exon 9 and its flanking introns across 100 vertebrates, related to Figure 1E. Human *GFPT1* is encoded on the opposite strand, and is indicated on the top. Exon 9 is indicated by a yellow box. The bindings sites for SRSF1, Rbfox, and hnRNP H/F are indicated in blue, red, and purple boxes, respectively. Amino acids and nucleotides are indicated for exon 9 and introns 8/9, respectively. Lack of a corresponding nucleotide is indicated by =.

Supplementary Figure S4, related to Figure 1

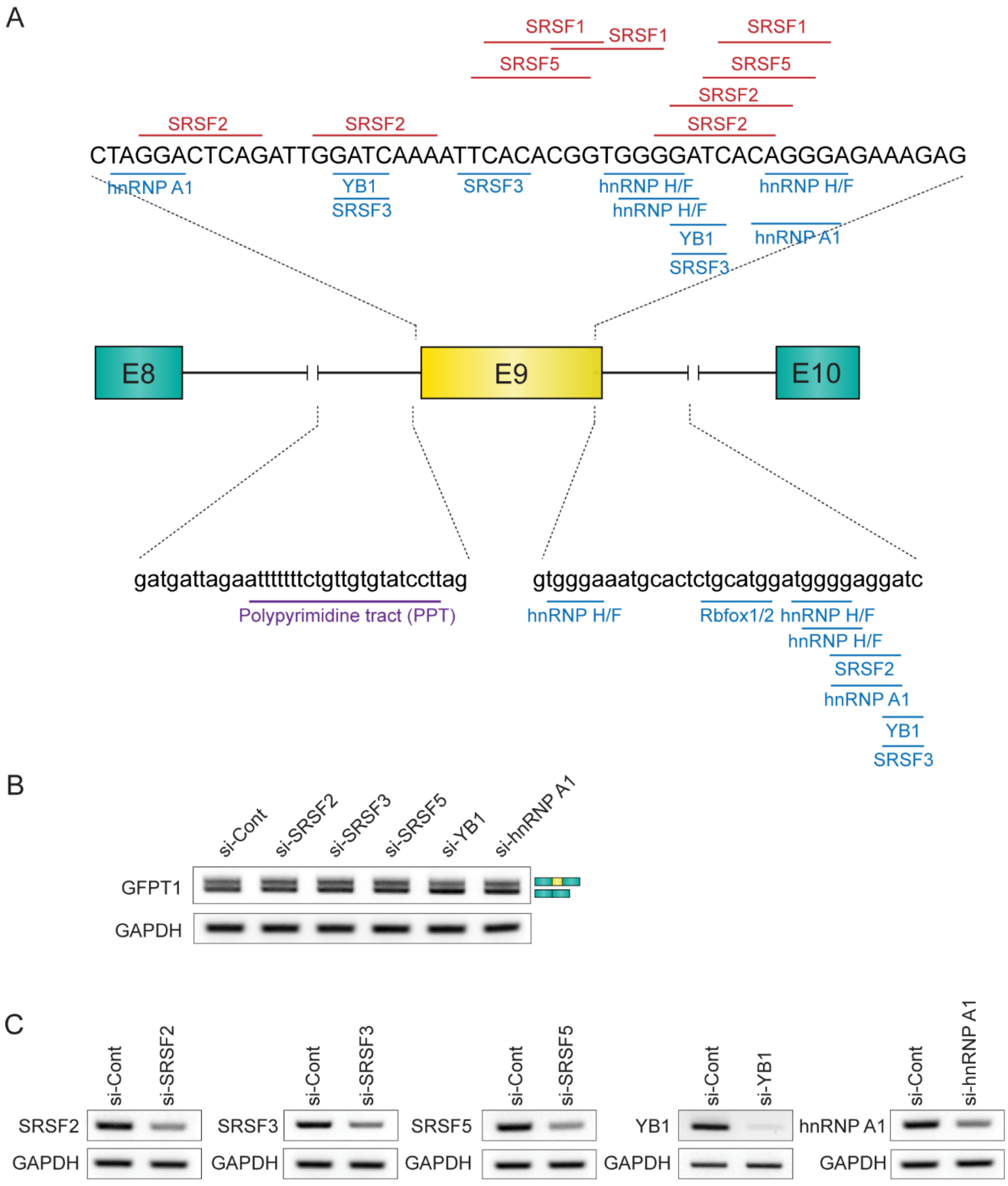

Supplementary Figure S5, related to Figure 2

Supplementary Figure S5. Motifs of RNA-binding proteins (RBPs) predicted by ESE Finder 3.0 and SpliceAid 2, related to Figure 2. **(A)** Schematic of human *GFPT1* gene spanning exons 8 to 10. Nucleotide sequences of *GFPT1* exon 9 and its flanking intronic regions are shown. ESE Finder 3.0 predicted binding motifs for SR proteins in exon 9 (shown in red letters and lines). SpliceAid 2 predicted additional binding motifs for other RBPs in exon 9 and intron 9 (shown in blue letters and lines). The putative polypyrimidine tract (PPT) at the 3' splice site is indicated in purple letters and lines. **(B)** RT-PCR of endogenous *GFPT1* in differentiated KD3/Hu5 myotubes after knockdown with siRNAs against control (si-Cont), *SRSF2* (si-SRSF2), *SRSF3* (si-SRSF3), *SRSF5* (si-SRSF5), *YBX1* (si-YB1), and *HNRNPA1* (si-hnRNP A1). Splice variants are schematically shown on the right side. **(C)** Confirmation of siRNA-mediated knockdown of each RBP by RT-PCR. Expression of *GAPDH* is shown as an internal control.

Supplementary Figure S5, related to Figure 2

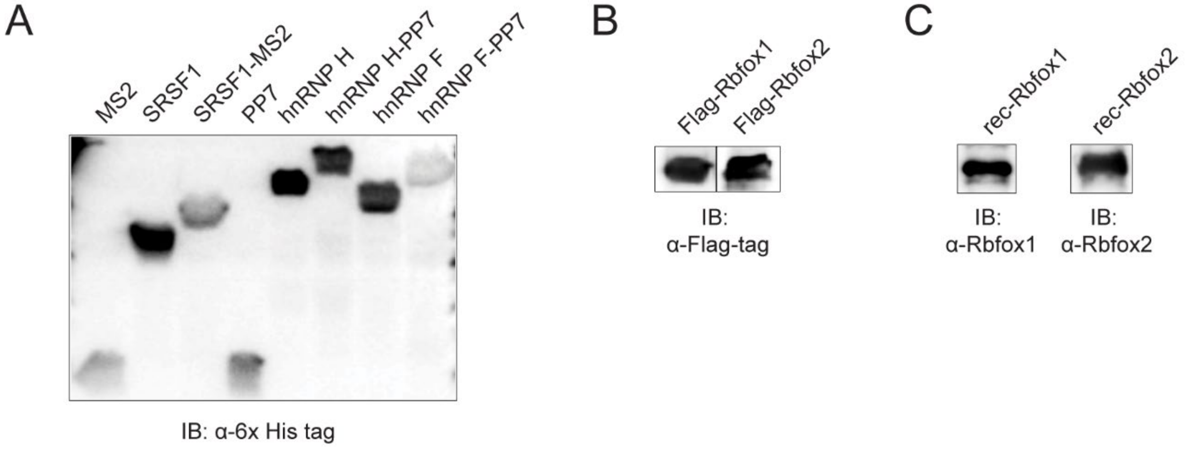

Supplementary Figure S6. Confirmation of the expression of RNA-binding proteins (RBPs), related to STAR Methods. (A, B) Immunoblotting of indicated RBPs transfected into HeLa cells with anti-6xHis-tag antibody **(A)** and anti-Flag-tag antibody **(B)**. **(C)** Immunoblotting of recombinant human Rbfox1 and Rbfox2 in *E. coli* with anti-Rbfox1 and anti-Rbfox2 antibodies.

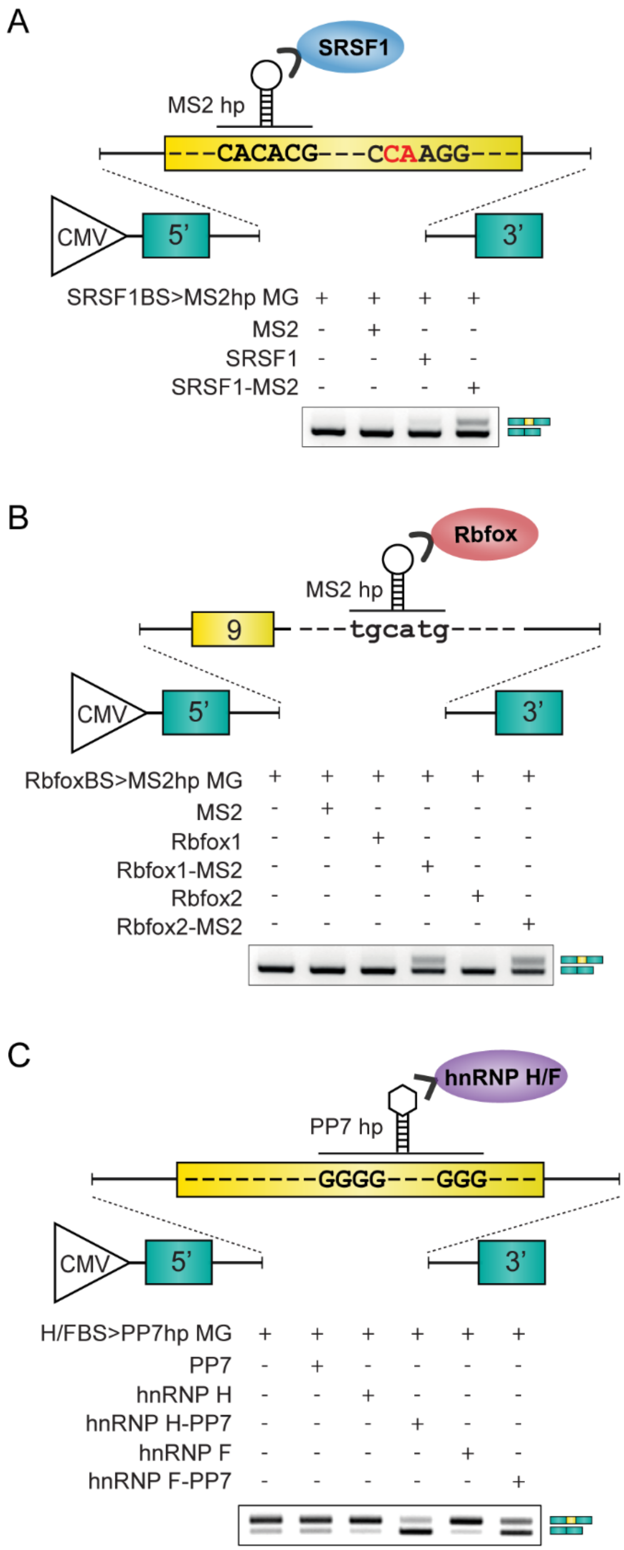

Supplementary Figure S7, related to Figure 2

Supplementary Figure S7. Position-specific splicing regulatory effects of SRSF1, Rbfox1/2, and hnRNP H/F on the identified *cis*-elements, related to Figure 2. (A) Schematic of the *GFPT1* minigene carrying the MS2 hairpin-loop (hp) substituting for the first SRSF1-binding motif in exon 9, while the second putative SRSF1-binding motif was mutated (red letters). SRSF1 was fused to the MS2 coat protein (inverted U-shaped) to directly tether SRSF1 to the MS2 hairpin-loop. **(B)** Schematic of the *GFPT1* minigene carrying the MS2 hairpin-loop substituting for the Rbfox-binding motif in intron 9. Rbfox1 and Rbfox2 were fused to the MS2 coat protein. **(C)** Schematic of the *GFPT1* minigene carrying the PP7 hairpin-loop substituting for the hnRNP H/F-binding motifs in exon 9. hnRNP H and F were fused to the PP7 coat protein. **(A, B, C)** RT-PCR of minigenes cotransfected with the effector cDNAs in KD3/Hu5 myoblasts.

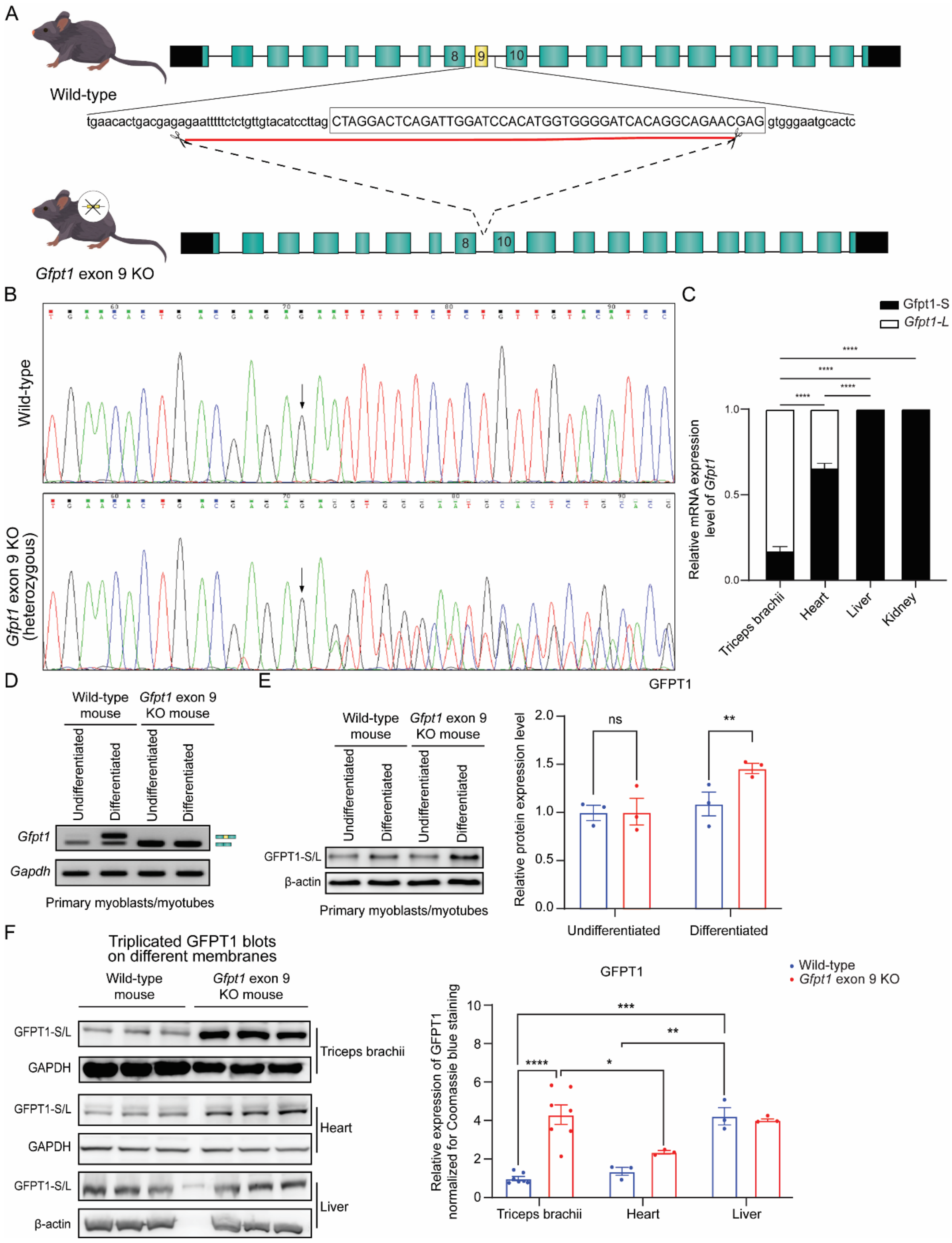

Supplementary Figure S8, related to Figure 4

Supplementary Figure S8. Generation of *Gfpt1* exon 9 KO mice, related to Figure 4. **(A)** Schematic of the genomic structure of mouse *Gfpt1* gene. Exons are shown in boxes and introns are shown in solid lines. The 5' and 3' untranslated regions (UTRs), constitutive exons, and alternatively spliced exon 9 are indicated by black, blue, and yellow boxes. Exon 9 and its flanking intronic nucleotides are shown in uppercase and lowercase letters, respectively. Artificial deletion of 72 nucleotides in intron 8 and exon 9 in *Gfpt1* exon 9 KO mice is underlined in red. **(B)** Sequencing chromatograms showing the 72-bp deletion in heterozygous *Gfpt1* exon 9 KO mouse. Arrows point to the 5' end of the 72-nt deleted segment. **(C)** Relative mRNA expression levels of *Gfpt1-S* and *Gfpt1-L* isoforms in indicated tissues in wild-type mice (n = 3 mice each). **(D)** Representative RT-PCR of endogenous *Gfpt1* in undifferentiated and differentiated primary myoblasts isolated from wild-type and *Gfpt1* exon 9 KO mice. **(E)** Representative immunoblots and quantification of endogenous Gfpt1 (both short and long isoforms, S/L) in undifferentiated and differentiated primary myoblasts isolated from wild-type and *Gfpt1* exon 9 KO mice. **(F)** Representative immunoblots and quantification of endogenous Gfpt1 (both short and long isoforms, S/L) in triceps brachii, heart, and liver of wild-type and *Gfpt1* exon 9 KO mice at age 12 months (n = 3 mice each). The amount of GFPT1 was normalized for the total protein estimated by Coomassie blue staining, and also for that of wild-type triceps brachii muscle. **(C**, **E**, **F**, and **G)** Mean and SEM are indicated. Two-way ANOVA with posthoc Tukey test was applied to **E**, **F**, and **G.** One way ANOVA with posthoc Tukey test was applied to **C.** $^*p < 0.05$, $^{**}p < 0.01$, $^{***}p < 0.001$, and $^{****}p < 0.0001$.

Supplementary Figure S8, related to Figure 4

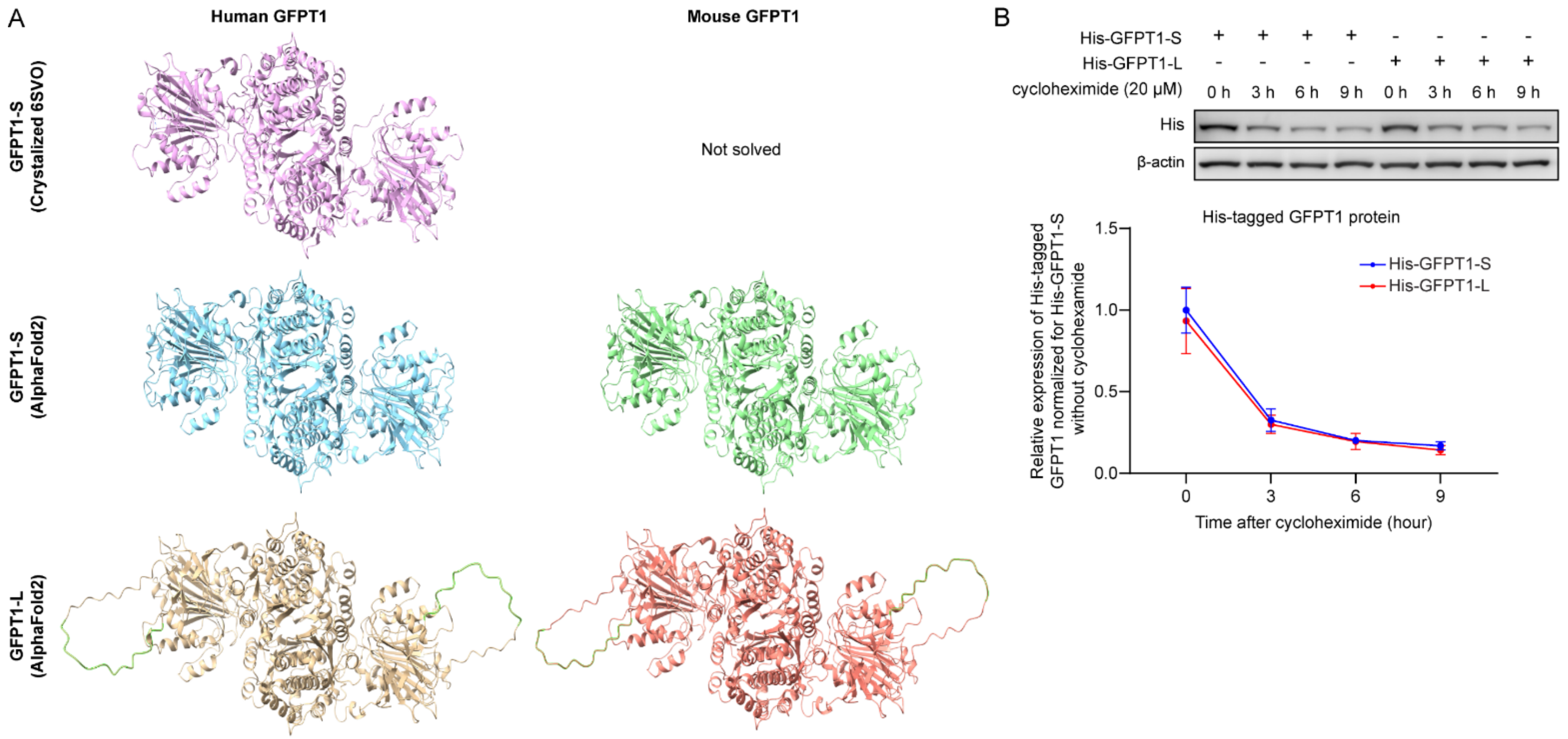

Supplementary Figure S9. Solved and AlphaFold2-predicted structures of human and mouse GFPT1-S and -L, related to Figure 4. (A) Dimeric structures of human and mouse GFPT1-S and -L were predicted by AlphaFold2 using ColabFold[99], and were aligned to the solved crystal structure of dimeric human GFPT1-S in complex with glucosamine-6-phosphate and L-glutamate (PDB ID: 6SVO)[100]. Amino acids encoded by exon 9 are highlighted in green in the loops of human and mouse GFPT1-L. Loops represent disorganized structures, or AlphaFold2 failed to predict their structures. **(B)** Representative immunoblots and quantification of temporal profiles of transfected His-tagged GFPT1 protein in HEK293 cells. The cells were harvested at the indicated time points after adding cycloheximide. Mean and SD are indicated (n = 3). No statistical significance by two-way ANOVA.

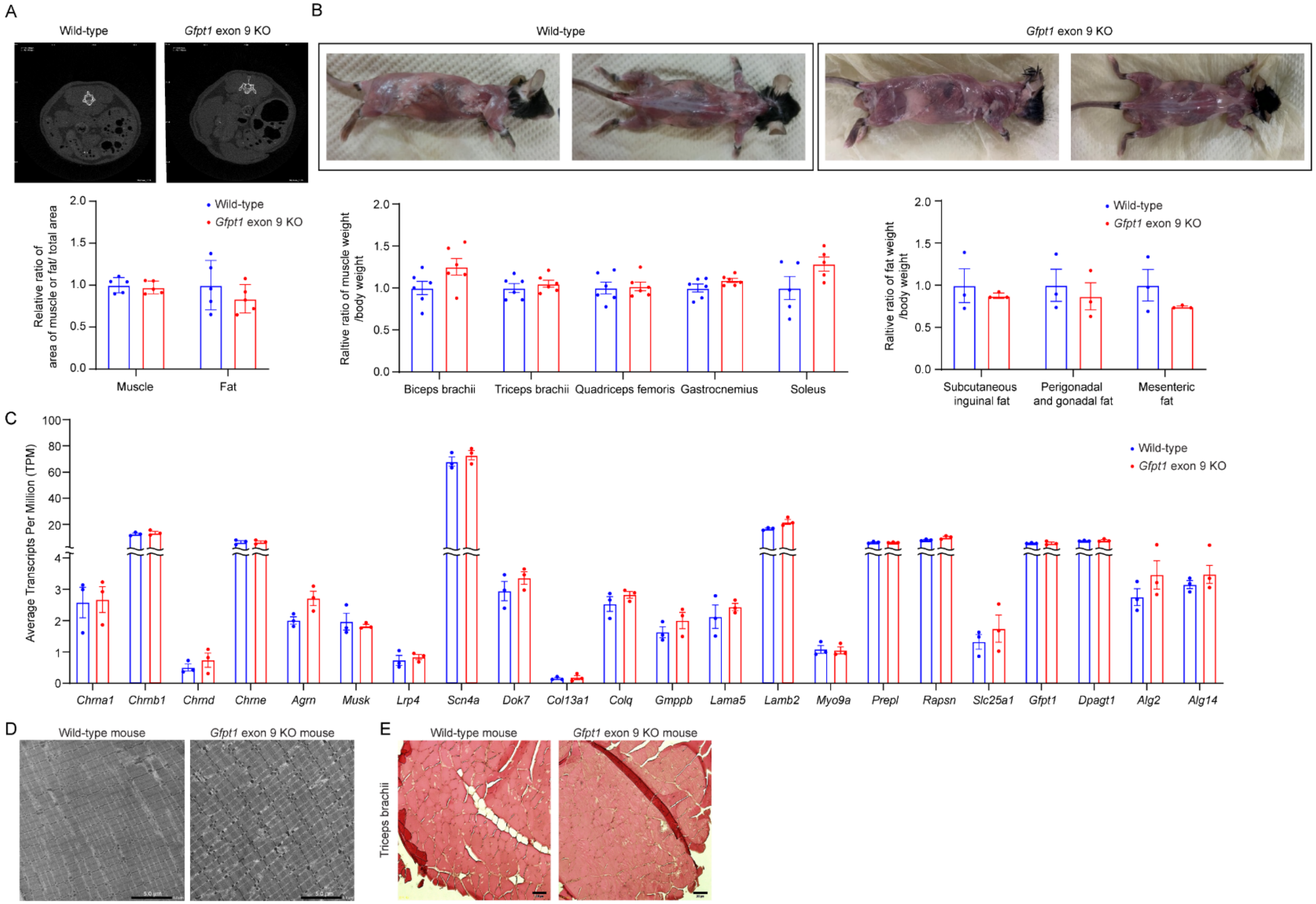

Supplementary Figure S10, related to Figure 5

Supplementary Figure S10 related to Figure 5. ***Gfpt1*** **exon 9 KO mice showed normal muscle mass and body fat, related to Figure 5**. **(A)** Representative micro-computed tomography (µCT) images. The areas of paravertebral muscles and fat were divided by the total cross-sectional area, and also by the ratio of wild-type mice (*n* = 5 mice each). **(B)** Representative images of subcutaneous fats and muscles. The wet weight of indicated muscle (left) or fat (right) was divided by the body weight, and also by the ratio of wild-type mice (*n* = 3 mice each). **(C)** RNA-seq analysis of triceps brachii of wild-type and *Gfpt1* exon 9 KO mice at age 13 months (*n* = 3 mice each). Twenty-two representative genes at the NMJ, some of which are mutated in CMS, were analyzed. **(D)** Representative electron micrographs of the triceps brachii in wild-type and *Gfpt1* exon 9 KO mice. Scale bar = 5 µm. **(E)** Representative triceps brachii muscles stained with hematoxylin and eosin in wild-type and *Gfpt1* exon 9 KO mice. Scale bar = 200 µm. **(A**, **B**, and **C)** Mean and SEM are indicated. No statistical significance by two-way ANOVA in **A**, **B**, and **C**.

Supplementary Table S1. Primer sequences for PCR, RT-PCR, and qRT-PCR, related to STAR Methods

Primers	5'-3' sequences
**Primers for RT-PCR of endogenous human *GFPT1***	
GFPT1-E8/F	AGCCCTCTGTTGATTGGTGT
GFPT1-E10/R	TGCTTTTTCTTCCACCGGGA
**Primers for RT-PCR of pSPL3-*GFPT1* minigene**	
pSPL3-3'E/F	TCTGAGTCACCTGGACAACC
pSPL3-3'E/R	ATCTCAGTGGTATTTGTGAGC
**Primers for RT-PCR of human molecules**	
hMyoG/F	GGTGCCCAGCGAATGC
hMyoG/R	TGATGCTGTCCACGATGGA
hMyoD1/F	ACGTGAGGACGAGCATGTG
hMyoD1/R	GGTTTGGATTGCTCGACGTG
hGAPDH/F	ATGGCACCGTCAAGGCTGAGA
hGAPDH/R	GGCATGGACTGTGGTCATGAG
hSRSF2/F	CACTCGTTCTCGATCTCGGTC
hSRSF2/R	TCGATCGCGACCTGGATTTG
hSRSF3/F	GGAACGGGCTTTTGGCTACT
hSRSF3/R	ACGGCAGCCACATAGTGTTC
hSRSF5/F	GAGACTAAATCCAGCGGCCA
hSRSF5/R	GACCGAGCCCTAGCATGTTC
hYB1/F	GTCATCGCAACGAAGGTTTT
hYB1/R	AACTGGAACACCACCAGGAC
hHnRNP A1/F	GGAGGTGGATGCAGCTATGA
hHnRNP A1/R	CAGTGTCTTCTTTAATGCCACCA
**Primers for both RT-PCR and qRT-PCR of RBPs**	
qRT-SRSF1/F	CCAGACATCCGAACCAAGGA
qRT-SRSF1/R	GCAGACGGTACCCATCGTAA
qRT-Rbfox1/F	CGGTTCCCGAGCACACATTA
qRT-Rbfox1/R	CCCGGAACCTGAAGGGGATA
qRT-Rbfox2/F	GCAGCCTACAGATATGCACAG
qRT-Rbfox2/R	GCTGTAGCCACCTCGGTATAA
qRT-hnRNP H/F	TAGTCCTGACACGGCCAATG
qRT-hnRNP H/R	CCTGTGAAGCAAACTGCACG
qRT-hnRNP F/F	TCCCACAGAACCGAGATGGA
qRT-hnRNP F/R	CACGAACGCTTCCCCTGTAA
**Primers for RT-PCR of mouse molecules**	
mGfpt1-E8F	AGCCCTCTCTTGATTGGTGT
mGfpt1-E10R	CAACTGCCTTTTCCTCAACAG
real time-m-Gapdh-F (PMID: 18070882)	GTTGTCTCCTGCGACTTCA
real time-m-Gapdh-R (PMID: 18070882)	TGCTGTAGCCGTATTCATTG
**Primers for mouse genotyping**	
mGfpt1_Int8_F	ATATGCTGCAGTGAATGTTCTCCG
mGfpt1_Int9_R	TCGAAATGGCAAAAGCAAACAGAC
**Primers for off-targets**	
Chr 9-F (off-target chr9: 45105637-45105659)	TGACAGGAGCAAACACAAGCCC
Chr 9-R	GAGACTCAGAGAAGAAAAGCACCCAG
Chr 11-F (off-target chr11: 96056967-96056989)	GGGGCTAGGGTCCAAACATGG
Chr 11-R	AGAAGAAAACCTCTATCTCCGCACCTG

Chr 18-F (off-target chr18: 9354314-9354336)	TGCTAACAGAGTAGAACACTGGGTCA
Chr 18-R	ACAATCATAAGGGCACCTGCTG
Chr 1-F (off-target chr1: 42687861-42687883)	CCACCACTAAACTACTTGATGGAAGTCT
Chr 1-R	AATAAGGCAAACACAGTCCTCAGC
Chr 13-F (off-target chr13: 49306493-49306515)	AGTCGCCCTTTTGCTATGCCA
Chr 13-R	ACGATGGACTGAGGGCAAACTG
Chr 7-F (off-target chr7: 127391011-127391033)	AGGGGGCTGCTTCAATAGGAATG
Chr 7-R	AGAAAAGGAGAGAGTATGTGGGGTG
Chr 8-F (off-target chr8: 11116343-11116365)	GCTTGACTTGGAGAGCCATTCC
Chr 8-R	AAGCAAACAAGCCCCAGGTCAC
Chr 14-F (off-target chr14: 65075148-65075170)	CTTACTGGAAGCAGAAAGCCAATG
Chr 14-R	TTAGGAGTGAAGCCTTAGTGGAGG

Supplementary Table S2. Metabolomic analysis of triceps brachii muscles by capillary electrophoresis-mass spectrometry (CE-MS), related to Figure 4

Metabolites	Wild-type (*n* =3 mice)		Gfpt1 exon 9 KO (*n* =3 mice)		*Gfpt1* exon 9 KO / Wild-type		
	Mean	SD	Mean	SD	Ratio	*p*-value[a]	
2-Hydroxyglutaric acid	9.5	1.7	12	2.9	1.3	0.218	
2-Oxoisovaleric acid	8	2.4	5.2	0.3	0.6	0.174	
6-Phosphogluconic acid	5.2	0.4	4.6	1.9	0.9	0.638	
Acetyl CoA	0.7	0.2	0.9	0.2	1.4	0.133	
Adenine	3.7	0.4	4.2	0.3	1.2	0.136	
Adenosine	5.8	2.1	10	2.8	1.8	0.089	
Adenylate Energy Charge	1	0.014	1	0.014	1.0	0.977	
Adenylosuccinic acid	16	13	15	6.1	0.9	0.847	
ADP	309	107	322	147	1.0	0.908	
ADP-ribose	8.8	2.6	8	2.5	0.9	0.715	
Ala	2,019	346	1,858	101	0.9	0.509	
AMP	18	18	9.8	2.8	0.5	0.497	
Arg	137	15	101	22	0.7	0.081	
Argininosuccinic acid	4.6	0.7	3.6	0.2	0.8	0.125	
Asn	98	18	73	18	0.7	0.166	
Asp	328	61	170	43	0.5	0.025	*
ATP	5,162	328	4,966	199	1.0	0.437	
cAMP	0.4	0.08	0.3	0.06	0.9	0.533	
Carnitine	150	19	125	11	0.8	0.141	
Carnosine	3,775	244	3,472	488	0.9	0.409	
Choline	65	32	23	24	0.4	0.274	
cis-Aconitic acid	0.4	0.12	0.3	0.15	0.8	0.512	
Citric acid	103	13	81	14	0.8	0.127	
Citrulline	89	8.9	66	5.7	0.7	0.029	*
Citrulline/Ornithine	5.7	0.2	5	0.6	0.9	0.147	
CoA	2	0.8	2.2	0.4	1.1	0.803	
Creatine	18,883	609	18,863	593	1.0	0.969	
Creatinine	61	2.2	61	4.8	1.0	0.927	
Dihydroxyacetone phosphate	52	26	58	18	1.1	0.758	
Fischer's Ratio	3.1	0.4	2.4	0.8	0.8	0.263	
Fructose 1-phosphate	39	4.7	32	7.7	0.8	0.279	
Fructose 6-phosphate	249	33	216	25	0.9	0.238	
Fumaric acid	143	8	130	27	0.9	0.493	
G6P/R5P	1,326	127	1,138	622	0.9	0.656	
Galactose 1-phosphate	5.7	1.3	4.1	0.8	0.7	0.177	
GDP	5.4	3	5.7	2.6	1.0	0.921	
Gln	1,394	152	1,406	252	1.0	0.947	
Glu	1,084	59	836	281	0.8	0.264	
Glucose 1-phosphate	62	14	47	7.5	0.8	0.190	
Glucose 6-phosphate	596	70	505	65	0.8	0.176	
Glutathione (GSH)	391	42	320	70	0.8	0.220	
Glutathione (GSSG)	85	4.9	80	6.1	0.9	0.321	
Gly	2,268	547	1,636	166	0.7	0.176	
Glycerol 3-phosphate/DHAP	17	11	14	5	0.8	0.748	
Glycolic acid	3.4	1.4	2.3	0.9	0.7	0.349	

GMP	2.5	2	1.8	0.2	0.7	0.632	
GSH/GSSG	4.6	0.2	4.1	1.1	0.9	0.506	
GTP	128	1.7	128	15	1.0	0.970	
Guanosine	2.4	0.4	2.3	0.02	1.0	0.866	
Guanylate Energy Charge	1	0.02	1	0.014	1.0	0.903	
His	127	8.3	109	16	0.9	0.186	
Hydroxyproline	107	41	74	14	0.7	0.287	
Hypoxanthine	6.7	1	7.4	0.9	1.1	0.426	
Ile	134	25	96	21	0.7	0.118	
IMP	515	401	485	75	0.9	0.910	
Inosine	22	10	29	4.8	1.3	0.404	
Lactate/Pyruvate	115	12	141	15	1.2	0.091	
Lactic acid	18,462	2,729	18,746	822	1.0	0.877	
Leu	208	35	147	33	0.7	0.093	
Lys	379	31	280	69	0.7	0.117	
Malate/Asp	1.1	0.15	1.7	0.03	1.5	0.021	*
Malic acid	367	23	283	72	0.8	0.172	
Malonyl CoA	0.5	0.07	0.4	0.04	0.8	0.186	
Met	216	29	169	22	0.8	0.098	
N,*N*-Dimethylglycine	5.3	0.6	5.6	1.3	1.1	0.747	
N-Acetylglutamic acid	1	0.13	1	0.03	1.0	0.754	
NAD^+	390	20	413	9.5	1.1	0.162	
NADH	9.1	0.4	9.8	1.8	1.1	0.598	
NADH/NAD+	0.02	0.002	0.02	0.004	1.0	0.966	
$NADP^+$	7.6	0.6	8.9	0.7	1.2	0.071	
NADPH	3.9	0.6	3.4	0.5	0.9	0.314	
NADPH/NADP+	0.5	0.11	0.4	0.06	0.7	0.149	
N-Carbamoylaspartic acid	0.05	0.008	0.03	0.013	0.6	0.092	
Ornithine	15	1.1	13	1.1	0.9	0.071	
Phe	84	11	73	13	0.9	0.316	
Phosphocreatine	596	292	609	61	1.0	0.949	
Phosphoenolpyruvic acid	1.3	0.2	1.4	0.7	1.1	0.757	
Pro	317	58	213	32	0.7	0.070	
PRPP	22	2.3	26	7.3	1.2	0.500	
Pyruvic acid	160	17	134	12	0.8	0.102	
Ribose 1-phosphate	6.1	1.4	8.2	0.7	1.3	0.102	
Ribose 5-phosphate	0.5	0.07	0.5	0.2	1.1	0.658	
Ribulose 5-phosphate	1	0.4	1.4	0.3	1.5	0.220	
S-Adenosylhomocysteine	4.4	0.7	3.9	0.7	0.9	0.383	
S-Adenosylmethionine	17	1.2	12	1.5	0.7	0.016	*
SAM/SAH	3.8	0.3	3.2	0.3	0.8	0.071	
Sarcosine	4.1	0.3	3.9	1	1.0	0.793	
Ser	247	29	212	62	0.9	0.445	
Thr	261	25	184	10	0.7	0.022	*
Total Acetyl CoA-related Amino Acids	745	91	552	24	0.7	0.059	
Total Adenylate	5,489	207	5,298	171	1.0	0.288	
Total Amino Acids	9,688	1,239	7,875	251	0.8	0.122	
Total Aromatic Amino Acids	197	12	188	37	1.0	0.718	
Total BCAA	616	108	440	91	0.7	0.100	
Total Essential Amino Acids	1,706	209	1,283	69	0.8	0.061	
Total Fumarate-related Amino Acids	173	8.8	160	29	0.9	0.508	

Total Glucogenic Amino Acids	9,102	1,192	7,448	244	0.8	0.133	
Total Glu-related Amino Acids	3,059	163	2,664	112	0.9	0.031	*
Total Glutathione	561	52	479	58	0.9	0.144	
Total Guanylate	136	6.6	135	13	1.0	0.929	
Total Ketogenic Amino Acids	1,179	122	895	61	0.8	0.038	*
Total Non-essential Amino Acids	7,983	1,060	6,592	186	0.8	0.147	
Total Oxaloacetate-related Amino Acids	427	75	243	25	0.6	0.041	*
Total Pyr-related Amino Acids	4,819	903	3,919	149	0.8	0.224	
Total Succinyl CoA-related Amino Acids	624	102	462	60	0.7	0.094	
Trp	24	3.1	29	8.1	1.2	0.435	
Tyr	90	3.7	87	17	1.0	0.819	
UDP-glucose	11	0.7	13	1.6	1.2	0.092	
Urea	7,998	818	4,353	611	0.5	0.004	**
Uric acid	5	0.4	6.3	0.6	1.3	0.042	*
Val	274	48	197	38	0.7	0.102	
Xanthine	2.6	0.7	3.4	0.5	1.3	0.217	
β-Ala	97	8.1	69	18	0.7	0.098	
γ-Aminobutyric acid	3.2	0.3	4.6	1.2	1.5	0.175	

[a]Welch's *t*-test (*$p < 0.05$ and **$p < 0.01$).

Supplementary Data S1. Python script to measure AChR area, related to Figure 5

```
from PIL import Image
import numpy
from pylab import *
import glob
plt.rcParams.update({'figure.max_open_warning': 0})

# LOAD THE IMAGE TO BE ANALYZED
path='AChR-Tri/WT/13M#3'

image_names=glob.glob(path+'/*.tif')

threshold=50 # # CHANGE HERE, choose this based on the image
savepng=True
savetxt=True
############

if savetxt:
    output_txt='Result_'+path.replace('/','_')+'.txt'
    print('Saving result as txt file to ... '+output_txt)
    txt_file=open(output_txt,'w')
    txt_file.write('name \t area\n')

for image in image_names:
    im=Image.open(image)

    imarray=numpy.array(im)     # convert to numpy array
    figure(figsize=(10,4))
    subplot(1,2,1)
    imshow(imarray,cmap='hot')
    title(image)
    colorbar()

    # define the marked area
    binary=zeros(imarray[:,:,0].shape)
    idx=where(imarray[:,:,0]>threshold)
    binary[idx]=1
    area=binary.sum()

    # save to txt file
    if savetxt:
        txt_file.write(image+' \t %i\n' % area)

    # plot
    subplot(1,2,2)
    imshow(binary,cmap='gray_r')
    title('Area of an object = %i' % area)
    tight_layout()
    # show()
    # pause(1)
```

```
    if savepng:
        output_name=image.replace('tif','png')
        print('Saving plot as png file to ... '+output_name)
        savefig(output_name,format='png')

if savetxt:
    txt_file.close()
```
